# Non-Coding RNAs (microRNAs, lncRNAs, circRNAs) in Adenomyosis: A Systematic Review of Mechanistic and Translational Evidence

**DOI:** 10.3390/ijms262110713

**Published:** 2025-11-04

**Authors:** Rafał Watrowski, Stoyan Kostov, Mario Palumbo, Andrea Rosati, Radmila Sparić, Ibrahim Alkatout, Ingolf Juhasz-Böss, Salvatore Giovanni Vitale, Liliana Mereu

**Affiliations:** 1 Department of Gynecology, Helios Hospital Müllheim, Teaching Hospital of the University of Freiburg, Heliosweg 1, 79379 Müllheim, Germany; 2 Faculty of Medicine, University of Freiburg, 79106 Freiburg, Germany; 3 Research Institute, Medical University Pleven, 5800 Pleven, Bulgaria; drstoqn.kostov@gmail.com; 4 Department of Gynecology, Hospital “Saint Anna”, 9002 Varna, Bulgaria; 5 Department of Public Health, School of Medicine, University of Naples Federico II, 80138 Naples, Italy; mpalumbo@gmail.com; 6 Department of Woman’s and Child Health and Public Health Sciences, Gynecologic Oncology Unit, Fondazione Policlinico Universitario Agostino Gemelli IRCCS, 00168 Rome, Italy; dott.andrearosati89@gmail.com; 7 Faculty of Medicine, University of Belgrade, Dr Subotića 8, 11000 Belgrade, Serbia; radmila@rcub.bg.ac.rs; 8 Clinic for Gynecology and Obstetrics, University Clinical Centre of Serbia, Dr Koste Todorovića 26, 11000 Belgrade, Serbia; 9 Department of Obstetrics and Gynecology, University Hospitals Schleswig-Holstein, Campus Kiel, Arnold-Heller-Strasse 3 (House C), 24105 Kiel, Germany; ibrahim.alkatout@uksh.de; 10 Department of Obstetrics and Gynecology, Medical Center—University Hospital Freiburg, 79106 Freiburg, Germany; ingolf.juhasz-boess@uniklinik-freiburg.de; 11 Obstetrics and Gynecology Unit, Department of General Surgery and Medical Surgical Specialties, University of Catania, 95124 Catania, Italy; vitalesalvatore@hotmail.com (S.G.V.); liliana.mereu@unict.it (L.M.)

**Keywords:** adenomyosis, non-coding RNA (ncRNA), microRNA (miRNA), long non-coding RNA (lncRNA), circular RNA (circRNA), extracellular vesicles (EV), epigenetic regulation, biomarkers, diagnostic, translational, endometrial–myometrial interface

## Abstract

Adenomyosis (AM) is a hormonally responsive uterine disorder defined by ectopic endometrial tissue within the myometrium, causing pain, abnormal bleeding, and subfertility. Non-coding RNAs (ncRNAs)—including microRNAs (miRNAs), long non-coding RNAs (lncRNAs), and circular RNAs (circRNAs)—are post-transcriptional regulators implicated also in uterine remodeling. We systematically reviewed original studies evaluating ncRNAs in AM using human samples, in vitro and animal models, or bioinformatic approaches. Data sources included PubMed and Google Scholar (inception up to 10 August 2025). Forty-one studies were included and synthesized across mechanistic, diagnostic, and translational domains. miRNAs (*n* = 31) were the most studied subclass, followed by lncRNAs (*n* = 10) and circRNAs (*n* = 5). Recurrent miRNAs such as miR-10b and miR-30c-5p (downregulated, inhibitory) and miR-145 (upregulated, promotive) regulate epithelial invasion, epithelial–mesenchymal transition, and cytoskeletal remodeling via PI3K–AKT/MAPK and Talin1 signaling. The let-7a/LIN28B axis governed estrogen-sensitive proliferation in the junctional zone, while miR-21 exhibited compartment-specific roles in decidualization and ectopic cell survival. Extracellular-vesicle (EV)-bornemiRNAs (e.g., miR-92a-3p, miR-25-3p, miR-4669) contributed to immune polarization and show early diagnostic potential. lncRNAs and circRNAs acted via chromatin modifiers and ceRNA networks. Most findings remain at the discovery stage. Convergent dysregulation was observed in key signaling pathways, including JAK–STAT, Wnt/β-catenin, and Hippo–YAP. ncRNAs regulate critical axes of invasion, proliferation, immune modulation, and hormonal response in AM. Targets with preliminary causal support—miR-10b/ZEB1, let-7a/LIN28B, and miR-145/Talin1—warrant further validation. Circulating miRNAs—especially in EVs—offer promise for non-invasive diagnosis.

## 1. Introduction

Adenomyosis (AM) is a chronic, estrogen-dependent disorder defined by the presence of ectopic endometrial glands and stroma within the myometrium, typically accompanied by smooth muscle hypertrophy and hyperplasia [1]. Advances in high-resolution transvaginal ultrasound (TVUS) and magnetic resonance imaging (MRI) have shifted diagnosis from postsurgical histology toward non-invasive recognition across the reproductive lifespan [1,2]. Clinically, AM is associated with a broad spectrum of symptoms, including abnormal uterine bleeding, dysmenorrhea, chronic pelvic pain, dyspareunia, and subfertility or recurrent pregnancy loss. Pain is a particularly prominent and burdensome feature, often presenting in diverse constellations such as cyclic or chronic pelvic pain, deep dyspareunia, and severe dysmenorrhea [1,2,3]. Symptom severity does not always correlate with lesion extent or subtype [1,2]. Moreover, AM coexists with other uterine pathologies, in up to 80% with endometriosis [3] and in up to 50% of cases with leiomyomas [4], but also with congenital uterine anomalies [5,6] or acquired structural defects disrupting the endometrial–myometrial interface (EMI) like post-caesarean niche (isthmocele) [7].

Current pathogenetic models include invagination of the endometrial basalis into the myometrium, de novo metaplastic transformation of displaced Müllerian remnants, or aberrant differentiation of adult stem/progenitor cells, typically framed by tissue-injury-and-repair (TIAR) at the EMI [1,8,9]. The key anatomic substrate is the junctional zone (JZ)—the hormonally responsive ‘inner’ myometrium immediately beneath the basalis that modulates uterine peristalsis—where chronic hypercontractility and microtrauma can prime intramyometrial ingrowth [1,8,9]. Complementing TIAR, the EMI disruption hypothesis posits that repetitive or iatrogenic injury initiates hypoxia–inflammation loops, epithelial–mesenchymal transition (EMT), and stem-cell recruitment, thereby accelerating lesion formation [8,9]. Converging molecular evidence—steroid-receptor dysregulation, inflammatory mediators, extracellular-matrix remodeling, neuroangiogenesis, and fibrosis—supports adenomyosis as aberrant tissue remodeling rather than a static structural defect/purely structural anomaly [8,9].

### 1.1. Non-Coding RNAs (ncRNAs)

**Figure 1:**
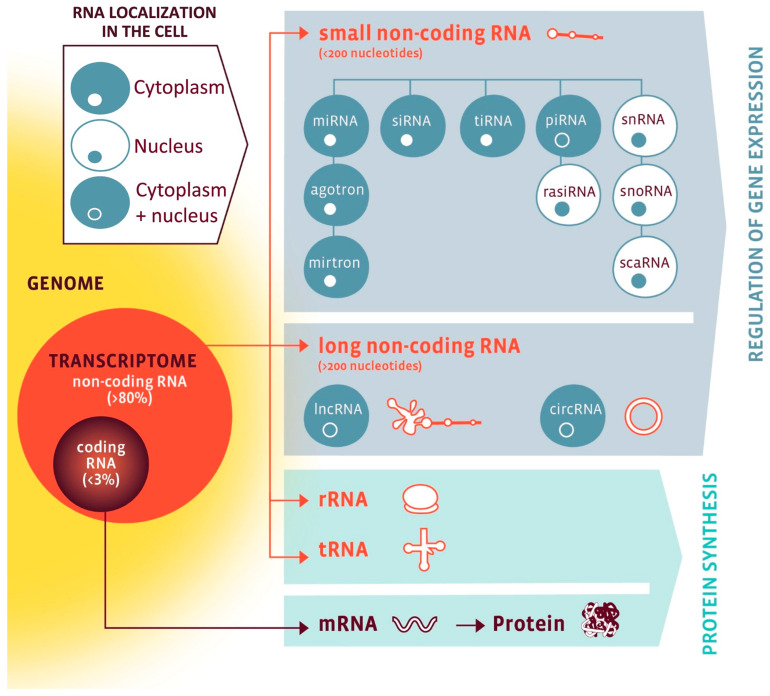
The RNA family. RNA classes are organized by coding potential (coding vs. non-coding), size (small vs. long), function (protein synthesis vs. gene regulation), and subcellular localization (nuclear, cytoplasmic, or both). Nuclear RNAs are shown in a white cell with blue nucleus; cytoplasmic RNAs in a blue cell with white nucleus; RNAs present in both compartments in a blue cell with a blue nucleus. Abbreviations: circRNA, circular RNA; lncRNA, long non-coding RNA; miRNA, microRNA; mRNA, messenger RNA; piRNA, PIWI-interacting RNA; rasiRNA, repeat-associated small interfering RNA; rRNA, ribosomal RNA; scaRNA, small Cajal body-specific RNA; siRNA, small interfering RNA; snoRNA, small nucleolar RNA; snRNA, small nuclear RNA; tiRNA, tRNA-derived stress-induced small RNA; tRNA, transfer RNA. Reproduced from Devaux et al., 2017 [12]. Open-access reuse under the article’s applicable Creative Commons license; no changes were made. Additional permission obtained via RightsLink.

While only 1–2% of the human genome (20,000–25,000 genes) encodes proteins, a much larger fraction is transcribed into ncRNAs with regulatory functions [10,11]. As visualized in Figure 1, ncRNAs are broadly classified into housekeeping species essential for protein synthesis, such as ribosomal RNAs (rRNAs), transfer RNAs (tRNAs), small nuclear RNAs (snRNAs), and small nucleolar RNAs (snoRNAs), as well as regulatory species, which include microRNAs (miRNAs), long non-coding RNAs (lncRNAs), circular RNA (circRNAs), Piwi-interacting RNAs (piRNAs), and small interfering RNAs (siRNAs) [12]. Importantly, both housekeeping and regulatory ncRNAs interact with other cellular components—and with each other—creating complex layers of coordinated gene regulation [10,11,13,14]. Their tissue specificity, dynamic regulation, and detectability in body fluids make them potentially suitable for molecular phenotyping and biomarker development [11,14,15,16,17].

#### 1.1.1. MicroRNAs (miRNAs)

miRNAs are short (20–24 nt), single-stranded RNAs that function as trans-acting regulators by binding complementary sequences in target mRNAs, typically suppressing translation. They associate with Argonaute proteins within the miRNA-induced silencing complex (miRISC), providing broad post-transcriptional control across hundreds of targets within a single family [13]. Within gene regulatory networks (GRNs), miRNAs act not merely as repressors but as robust modulators of developmental and homeostatic processes [13]. They can gate differentiation switches, buffer gene expression noise, and coordinate multi-gene modules. Many miRNAs are conditionally required—non-essential under baseline conditions but indispensable in stress or disease—highlighting their role as context-dependent stabilizers of cellular function [13].

In gynecology, miRNA dysregulation is implicated in conditions such as endometriosis [17,18,19], uterine fibroids [20], premature ovarian insufficiency [17], recurrent implantation failure [17], polycystic ovary syndrome (PCOS) [21,22], or gynecologic malignancies [14,15,16]. In these contexts, miRNAs modulate pathways being central also to AM pathogenesis, including inflammation, EMT, extracellular matrix (ECM) remodeling, angiogenesis, and key signaling cascades such as PI3K–AKT/mTOR, ERK/MAPK, JAK–STAT, Wnt/β-catenin, and Hippo–YAP [14,17,22,23].

Clinically, miRNAs are attractive candidates for non-invasive diagnostics due to their stability in biofluids (e.g., serum, plasma, saliva) and packaging in extracellular vesicles (EVs) [15,16,17,24]. The first commercially available test for endometriosis (EndoTest^®^) utilizes a panel of salivary miRNAs [25,26]. Although no such panel has yet been validated for AM, the translational framework is already in place.

#### 1.1.2. Long Non-Coding RNAs

LncRNAs are >500-nucleotide transcripts (earlier definitions: >200 nt) with little or no protein-coding potential [27]. They act as molecular signals, guides for chromatin-modifying complexes, decoys for RNA-binding proteins and microRNAs, and scaffolds for multi-component assemblies, thereby shaping gene regulation at transcriptional, post-transcriptional, and epigenetic levels [27,28]. lncRNAs show far greater tissue and cell-type specificity than protein-coding genes, making them particularly relevant in organ systems where tight spatial and temporal gene control is essential—such as the brain and reproductive tract [27,29]. Their genomic origins are diverse, including intergenic regions, enhancers, antisense strands of protein-coding genes, and introns, often overlapping or regulating adjacent loci [28]. Functionally relevant lncRNAs implicated in reproductive biology include HAND2-AS1, which is transcribed antisense to the HAND2 transcription factor and regulates placental differentiation and endometrial pathologies by modulating BMP signaling and acting as a miRNA sponge [30]; LINC-ROR, which sustains stemness in endometrial progenitor cells by sequestering miR-145 [31]; and TUG1, a lncRNA that can recruit PRC2/EZH2 to modulate gene expression and has been linked to proliferation, apoptosis, and—specifically in ovarian models—angiogenesis via LRG1 [32,33]. These mechanisms extend beyond oncogenesis to include embryonic patterning, hormone responsiveness, immune tolerance, and decidualization [29,34]. Their context-dependent actions, stability in biofluids, and disease-specific expression make lncRNAs attractive candidates for diagnostic biomarkers and therapeutic targets [33]. However, their functional complexity, typically low abundance, and reliance on highly specific RNA–protein interactions pose significant challenges for clinical translation [27].

#### 1.1.3. Circular RNAs (circRNAs)

Circular RNAs (circRNAs) are covalently closed, single-stranded RNA molecules produced by back-splicing of precursor transcripts—where a downstream 5′ splice donor joins an upstream 3′ splice acceptor—yielding loops that lack free 5′ and 3′ ends (and therefore a 5′ cap and 3′ poly(A) tail) and can originate from exonic, intronic, or exon–intron sequences [35,36,37]. This circular conformation confers resistance to exonuclease-mediated degradation, granting them significantly greater stability than most linear transcripts [35]. Once dismissed as splicing by-products, circRNAs are now recognized as functional regulators that can sponge miRNAs (classic example: CDR1as/ciRS-7 with >70 conserved miR-7 binding sites), bind or scaffold RNA-binding proteins, modulate transcription and alternative splicing, and—less commonly—encode bioactive micropeptides via IRES- or m^6^A-guided cap-independent translation [35,36,37]. They exhibit pronounced tissue- and developmental-stage specificity, with dynamic expression patterns linked to physiological remodeling and disease progression. While direct evidence in AM remains nascent, parallels from PCOS and endometriosis implicate circRNAs in estrogen signaling, EMT, and fibrotic pathways [22]. Emerging data position circRNAs as integral nodes within ncRNA regulatory networks that interface with lncRNAs and miRNAs, shaping uterine homeostasis and disease-associated remodeling [23,35,36]

### 1.2. Relevance to AM and Aim of the Review

Evidence indicates that ncRNAs intersect with several pathways central to adenomyosis (AM). First, they modulate endocrine responsiveness: miRNAs and lncRNAs influence estrogen/progesterone signaling and downstream effectors within the JZ and at the EMI [38,39]. Second, ncRNAs participate in inflammation and fibrosis through axes that involve NF-κB, TGF-β/Smad, ECM remodeling, and EMT-processes tied to pain, bleeding, and lesion stiffening [39]. Third, crosstalk among ncRNA classes (miRNAs, lncRNAs, circRNAs) and their exchange via EVs provides a regulatory layer linking epithelial–stromal interactions and microenvironmental signaling in AM [38]. The subsequent sections of this systematic review synthesize how ncRNAs interface with the well-described signaling cascades specifically in adenomyosis.

For translation, the stability of miRNAs, circRNAs, and selected lncRNAs in biofluids and their packaging in EVs supports the feasibility of non-invasive biomarker development, while clinical deployment still requires rigorous validation [23,38].

In this review, we synthesize current evidence on miRNAs, lncRNAs, and circRNAs in AM—covering pathogenesis, diagnosis, prognosis, and therapeutic potential—and translate molecular foundations into clinically relevant applications.

## 2. Materials and Methods

### 2.1. Eligibility Criteria

We included original research on miRNAs, lncRNAs, or circRNAs in AM, using human samples, experimental models (in vitro or animal), or bioinformatics/omics analyses, and clear reporting methods and results. We excluded reviews, conference abstracts without full data, studies unrelated to AM, and papers without primary ncRNA data. No language restrictions were applied. All inclusion and exclusion criteria were pre-specified before screening.

### 2.2. Information Sources and Search Strategy

We searched PubMed, the EBSCOhost platform (covering 37 databases), BASE (Bielefeld Academic Search Engine), and Google Scholar from database inception to 10 August 2025, and screened reference lists of relevant reviews and all included studies. One additional eligible article was identified by manual/citation searching.

For PubMed, we combined MeSH and free-text terms. The primary string was as follows:

(adenomyosis[MeSH Terms] OR adenomyosis[Title/Abstract]) AND ((“microRNA”[MeSH Terms] OR microRNA[Title/Abstract] OR miRNA*[Title/Abstract]) OR (“long noncoding RNA”[MeSH Terms] OR “long non-coding RNA”[Title/Abstract] OR lncRNA*[Title/Abstract]) OR (“circular RNA”[MeSH Terms] OR “circRNA”[Title/Abstract] OR “circular RNA”[Title/Abstract]))

Using the primary PubMed string above, 39 records were retrieved; 6 reviews and 2 non-substantive corrections were excluded at identification, leaving 31 candidate records from this string.

To maximize sensitivity and ensure capture of minimally indexed records, we conducted three supplementary free-text searches:

*Adenomyosis AND (miRNA OR microRNA)*—45 records, 9 excluded on title/abstract.

*Adenomyosis AND (lncRNA OR “long non-coding RNA”)*—13 records, 2 excluded on title/abstract.

*Adenomyosis AND (circRNA OR “circular RNA”)*—5 records, 0 excluded on title/abstract.

These three searches collectively retrieved 63 raw records; the total number of records identified from PubMed (*n* = 63) reflects the combined output of three complementary free-text PubMed searches, not an arithmetic sum of all search strings.

On EBSCOhost, we queried both the abstract (AB) and subject (SU) fields using the following:

*“AB (Adenomyosis AND (miRNA OR microRNA OR “non-coding RNA” OR circRNA OR “circular RNA” OR lncRNA))”*, and

*“SU (Adenomyosis AND (miRNA OR microRNA OR “non-coding RNA” OR circRNA OR “circular RNA” OR lncRNA))*”. These searches yielded 39 records

On BASE, we performed subject queries using the following:

*“subj:Adenomyosis AND subj:(miRNA OR microRNA OR “non-coding RNA” OR circRNA OR “circular RNA” OR lncRNA)”*, which returned 43 records.

For Google Scholar, we used the following:

*“adenomyosis” AND (“microRNA” OR “miRNA” OR “lncRNA” OR “long non-coding RNA” OR “circRNA” OR “circular RNA”)*

Searches were conducted without filters. This returned 3080 results. We screened entries in relevance order until no new eligible studies were identified within the last 500 consecutively reviewed records. Accordingly, 500 Google Scholar entries were screened; the remaining 2580 were recorded as “removed before screening–other reasons” in PRISMA. No date limits or filters were applied.

### 2.3. Selection Process

All records from PubMed, EBSCOhost, BASE, and Google Scholar were exported, merged, and deduplicated in Zotero prior to screening. Notably, some publications appeared across multiple queries due to dual investigation of ncRNA classes (e.g., ceRNA networks involving both circRNAs and miRNAs); these were retained as single entries after deduplication.

Two reviewers (R.W., S.K.) independently screened titles/abstracts and then full texts against the eligibility criteria. Disagreements were resolved by discussion; consensus was reached without a third-party adjudicator (S.G.V.). No automation tools were used for study selection or data extraction.

**Figure 2:**
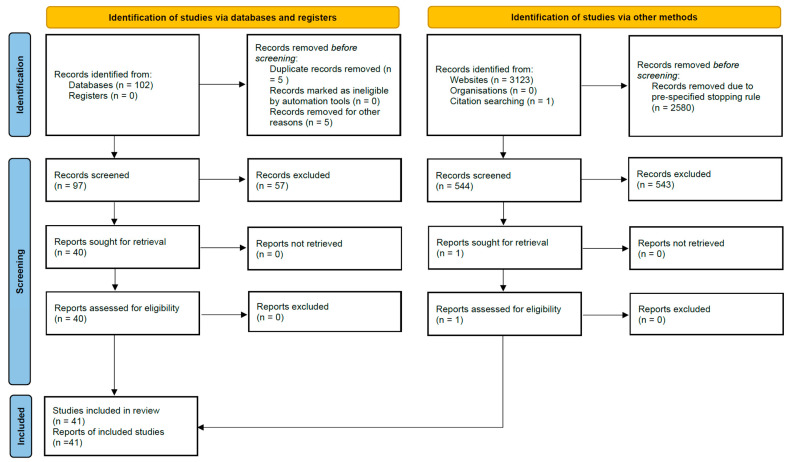
PRISMA 2020 flow diagram of study identification and selection. Records identified from Databases (*n* = 102; PubMed 63 + EBSCOhost 39), records identified from Websites (*n* = 3123; Google Scholar 3080 + BASE 43) and citation searching (*n* = 1). A pre-specified stopping rule led to 2580 Google Scholar records being removed before screening (500 Google Scholar and all 43 BASE records were screened).

After merging all search results, 63 records were identified from PubMed, 39 from EBSCOhost, 43 from BASE, and 500 from Google Scholar, plus 1 from citation searching, yielding 646 records before deduplication. A total of 5 duplicate records were removed during import, leaving 641 unique records for title/abstract screening. Full-text numbers (reports sought/assessed and exclusions with reasons) are shown in the PRISMA 2020 flow diagram (Figure 2); in total, 41 primary studies were included in the qualitative synthesis.

### 2.4. Data Items and Extraction

Three reviewers (R.W., S.K., M.P.) independently extracted data using a standardized form (piloted on five studies). Items included the following: study design; biological samples/compartments (eutopic endometrium, ectopic adenomyotic tissue, JZ and EMI, biofluids including serum/plasma/urine/vaginal secretions); cell types (EECs/ESCs, SMCs (JZ/EMI), eMSCs, macrophages, organoids); RNA type(s) (miRNA, lncRNA, circRNA); key molecules (targets/axes); techniques (qRT-PCR, RNA-seq/microarray, luciferase, RIP/ChIP, Western, EV TEM/NTA/markers); pathways (PI3K–AKT/mTOR, ERK/MAPK, Wnt/β-catenin, JAK–STAT, Hippo–YAP, TLR4/NF-κB, EMT); functional outcomes; clinical correlations; diagnostic metrics (AUC, sensitivity, specificity); interventions; confounders; validation (internal/external); and statistical methods. To prevent double-counting, studies examining multiple ncRNA classes were assigned a primary category (miRNA, lncRNA, circRNA) for synthesis, while cross-modality findings were recorded as secondary attributes.

### 2.5. Risk of Bias and Quality Appraisal

We used design-specific instruments for the quality assessment of included studies. For human observational studies (case-control, cross-sectional, pre/post tissue comparisons with an explicit comparator), we applied the Newcastle–Ottawa Scale (NOS) and mapped totals to High (≥7), Moderate (5–6), or Low (≤4) [40]. For animal experiments, we used the SYRCLE risk-of-bias tool [41]. For studies centered on EVs, we evaluated compliance with MISEV2018 (isolation method; particle characterization by TEM/NTA; positive/negative protein markers; dose normalization; uptake/functional assays; storage/reporting) [42]. For studies that developed or validated multivariable diagnostic/prognostic models (e.g., RNA panels or composite indices), we appraised reporting and analysis against TRIPOD (predictor definition/selection, handling of missing data, model building and internal/external validation, discrimination and calibration, and safeguards against overfitting) [43]. For descriptive high-throughput omics studies (without a prediction model), we applied an omics analysis checklist (normalization; multiple-testing correction/FDR; batch-effect assessment/mitigation; independent/orthogonal validation) [44]. For in vitro mechanistic work, we documented core rigor elements (cell identity/source/authentication, biological replicates, negative/positive controls, knock-down/over-expression efficiency and rescue specificity, and randomization/blinding of readouts where feasible) [44].

Each paper could be appraised by more than one instrument if multiple components were present (e.g., human tissue + in vitro; EV + omics). Two reviewers (R.W., S.G.V.) independently scored applicable domains; disagreements were resolved by consensus. We present, per study, the study type, the instrument(s) used, a final quality assessment (High/Moderate/Low with notes), and—where NOS applied—the item-level domain scores (Selection/Comparability/Outcome-Exposure) and total (0–9). For synthesis, we used a conservative rule: critical flaws in any applicable domain (e.g., EVs without particle/marker characterization; omics without FDR) downgraded the overall rating; otherwise, we reported the modal level with justification. Detailed appraisals are presented in the Quality Table (Supplementary Table S1).

### 2.6. Effect Measures and Synthesis Methods

Given heterogeneity in designs, samples, outcomes, and analytics, we conducted a narrative synthesis structured by pathogenesis, diagnostics, prognosis, and treatment. For diagnostic studies, we extracted AUC, sensitivity, specificity but did not pool estimates because of methodological diversity (compartments, assay platforms, thresholds, small samples). We summarized molecular axes/pathways, functional consequences, and clinical correlations, and produced three modality-specific tables (miRNA, lncRNA, circRNA) plus an EV/biofluid table.

### 2.7. Operational Definitions (Evidence Levels)

We introduced an operational evidence-level schema (L1–L3) for this review to distinguish association from causality and translational support. L1 (human association)—differential expression and/or clinical correlation in human samples (e.g., ROC/AUC; receiver operating characteristic/area under the curve; symptom links) without causal perturbation. L2 (in vitro mechanism)—causal gain/loss-of-function of the ncRNA or its direct target in relevant cells, ideally with rescue and target-engagement assays (e.g., dual-luciferase reporter with site mutagenesis; AGO-RIP/CLIP = Argonaute RNA immunoprecipitation/crosslinking immunoprecipitation; ChIP/ChIRP = chromatin immunoprecipitation/chromatin isolation by RNA purification), showing effects on disease-relevant phenotypes (proliferation, migration/EMT = epithelial–mesenchymal transition, apoptosis, contractility). L3 (in vivo/translation)—evidence from animal or xenograft models (e.g., antagomir/ASO = antisense oligonucleotide/shRNA = short hairpin RNA delivery; KO/KD = knockout/knockdown) and/or robust external validation of a diagnostic model. These criteria align with best-practice guidance for moving from association to mechanism (perturbation ± rescue/engagement) in ncRNA biology [45,46].

### 2.8. Reporting and Protocol

The review follows PRISMA 2020; the PRISMA checklist is provided in Supplement (Table S2). The review was not registered (no protocol registered in PROSPERO, which currently accepts only interventional studies); methods were pre-specified in an internal protocol (available on request).

### 2.9. Ethics

As a review of published data, ethics approval and consent were not required.

## 3. Results

### 3.1. Study Selection and Characteristics

We synthesized 41 primary studies examining the roles of ncRNAs in AM (Table 1) [47,48,49,50,51,52,53,54,55,56,57,58,59,60,61,62,63,64,65,66,67,68,69,70,71,72,73,74,75,76,77,78,79,80,81,82,83,84,85,86,87]. By subclass, miRNAs were the most frequently studied (*n* = 31) [47,48,49,52,57,58,59,60,61,62,63,64,65,66,67,69,70,72,73,74,76,77,78,79,80,81,83,84,85,86,87], followed by lncRNAs (*n* = 10) [50,51,53,55,56,59,66,71,75,82], and circRNAs (*n* = 5) [54,64,68,69,77]. Study designs varied: 32 studies utilized in vitro mechanistic models [48,49,50,52,53,54,55,56,57,58,59,60,61,62,63,64,65,66,67,68,69,70,72,74,75,79,80,81,82,83,84,85]; 24 involved human biopsy samples [48,49,50,53,54,55,58,59,61,62,63,66,67,68,71,73,74,75,77,80,82,83,86,87]; five used bioinformatic/omics [49,50,68,77,86], and only one employed an in vivo animal model (a Dicer knockout mouse, [47]). A total of seven studies investigated extracellular vesicle (EV) or biofluid compartments—including serum, plasma, urine, and vaginal secretions—indicating early translational exploration [73,74,76,78,84,85,87].

Biological compartments varied in focus. The majority of studies analyzed eutopic endometrial tissue, with fewer targeting ectopic lesions or the JZ, particularly the EMI. A small subset examined menstrual-phase differences, stromal–epithelial crosstalk, and macrophage-mediated immune microenvironments. Among functional readouts, proliferation was the most assessed outcome (*n* = 20) [52,53,59,60,61,62,63,64,66,67,69,70,71,72,74,75,79,80,82,83], followed by migration (*n* = 18) [48,56,59,63,64,65,67,69,70,71,72,74,75,80,81,82,83,85], invasion (*n* = 13) [48,56,59,64,65,67,69,70,71,75,80,85], EMT (*n* = 12) [48,56,59,63,64,65,69,71,72,78,81,84], apoptosis (*n* = 8) [52,59,62,69,75,79,80,83], angiogenesis (*n* = 3) [71,81,85], endometrial receptivity (*n* = 3) [54,55,73], autophagy (*n* = 2) [80,83], and macrophage polarization (*n* = 2) [78,84].

### 3.2. Convergent Pathways and Mechanistic Themes

Across RNA classes, a consistent set of signaling pathways recurred: PI3K–AKT–mTOR [48,60,83], MAPK/ERK [60,67], JAK–STAT [79], TLR4/NF-κB [59], Wnt/β-catenin [75], and Hippo–YAP [62]. These axes are often hormonally modulated—by estrogen via ERα (e.g., miR-145/CITED2 [81]; let-7a/LIN28B [60,61]) and by progesterone via LNG-responsive elements (e.g., H19/miR-17/TLR4 [59]). Pathways converge on epithelial invasion [48,64], stromal remodeling [63,75], immune polarization via EV cargo [78,84,87], and JZ proliferation [60,61], supporting a unifying model of AM as dysregulated tissue remodeling orchestrated by ncRNA networks across epithelial, stromal, immune, and vascular compartments. Across the corpus, sixteen studies functionally manipulated ncRNAs (overexpression/knockdown) and read out effects on downstream proteins or reporter constructs [48,56,60,61,62,63,64,65,67,69,70,71,72,75,79,83]; seven supported RNA–RNA or RNA–protein interactions (e.g., biotin pull-down, RIP, Ago2-IP, luciferase) [48,56,63,64,69,71,72]. Most studies integrated qRT-PCR validation, and subsets incorporated Western blotting (e.g., [60,79]), immunofluorescence (e.g., [61,62]), IHC (e.g., [49,50,51]), or EdU (e.g., [60,65,69]). Supplementary Table S3 details study designs and methodological features.

### 3.3. miRNAs: Functional Clusters and Compartment-Specific Actions

Several miRNAs showed downregulation in AM and convergence upon signaling pathways regulating epithelial invasiveness, EMT, and PI3K–AKT/MAPK activity. For instance, miR-10b directly targets ZEB1 and PIK3CA to restore E-cadherin and reduce p-AKT, thereby limiting migration/invasion (luciferase-validated, rescue confirmed) [48]. In contrast, miR-21 loss in eutopic glands is linked to impaired decidualization (HOXA10/IL-15 axis), with downstream infertility implications [57,80,83,85]. Conversely, miR-30c-5p suppression correlates with increased MAPK1 and proliferation [67], while miR-92a-3p (EV-enriched) correlates with reduced integrin-linked kinase and decreased stromal adhesion [85]. Tissue-level miRNA ratios (e.g., miR-93/miR-205, miR-20a/miR-17) also show diagnostic discrimination but require multi-center validation [58]. lncRNA–miRNA axes (e.g., H19/miR-17, MIR22HG/miR-2861) modulate PTEN and histone deacetylation pathways [59,66], whereas circRNAs (e.g., circPVT1) sponge miR-145-5p to release Talin-1, likely affecting focal adhesion dynamics [64,81]. EV-derived miRNAs (serum/urine/vaginal secretions) demonstrate diagnostic potential with AUCs up to 0.94, yet external validation is limited, and pre-analytical variability remains a concern [73,74,78,84,85,87].

In stromal and EMI compartments, miRNAs regulate tissue remodeling via JAK–STAT and EMT-related pathways. miR-124-3p, downregulated in AM, targets STAT3 and ZEB1, attenuating EMT markers and reducing invasiveness [48,63]; miR-145-5p activates Talin-1/focal-adhesion signaling in AM stroma [65,81]; miR-22/HDAC4 contributes to progesterone resistance in EMI, modulating apoptosis and proliferation [70].

JZ smooth muscle cells (SMCs) display hormone-sensitive proliferation through the let-7a/LIN28B axis. Overexpression of let-7a reduces LIN28B, p-AKT, and p-ERK and limits JZ-SMC proliferation; conversely, estrogen-induced LIN28B re-expression re-activates AKT–ERK signaling. The axis also interfaces with Hippo–YAP, potentially explaining JZ thickening and dysperistalsis [60,61,62].

Other compartment-specific miRNAs exhibit dual functions. In eutopic stroma, miR-21 promotes decidualization via suppression of RECK and TIMP3, whereas in epithelial compartments, miR-21 may act as an oncogenic driver in stroma under hormonal influence [57,80,83].

EV-based studies reveal upregulation of miR-92a-3p in serum-derived exosomes with diagnostic potential (AUC = 0.9435 in discovery; lower in validation), urinary exosomal signatures linked to fertility outcomes, and vaginal-fluid small-RNA panels that change after HIFU, all converging on adhesion/EMT pathways and subfertility associations [73,74,76,78,84,85].

### 3.4. lncRNAs: Chromatin Remodeling, ceRNA Networks, and Endocrine Regulation

lncRNAs act through both chromatin-associated mechanisms and ceRNA scaffolding. HAND2-AS1 regulates the HAND2–FGFR axis, affecting decidualization and JZ-SMC behavior; its silencing leads to FGF9 upregulation and increased proliferation/migration, driven by hypermethylation of its bidirectional promoter [82]. H19, acting as a ceRNA for miR-17, derepresses TLR4; its expression is modulated under levonorgestrel therapy [59]. Downregulation of MIR22HG and consequent hypomethylation-driven upregulation of miR-2861 are linked to impaired suppression of STAT3/MMP2 and increased epithelial proliferation [66]. TUG1, induced by EGR1, recruits EZH2 to silence TIMP2 via H3K27me3 methylation, thereby promoting epithelial invasion—a mechanism validated in two independent studies [56,71]. Silencing MIR503HG enhances stromal progression through miR-191/Wnt–β-catenin signaling [75].

Expression profiling studies revealed extensive lncRNA dysregulation in both eutopic and ectopic tissue compartments [50,51]. Comparative studies with endometriosis and recurrent implantation failure [55] showed overlapping lncRNA signatures, suggesting limited disease specificity but possible utility for endometrial receptivity assessment.

### 3.5. circRNAs: ceRNA Scaffolds and Pathway Modulation

circRNAs mainly function as miRNA sponges. circ_0061140 enhances LIN28B expression and epithelial proliferation by sponging miR-141-3p [69], integrating with the let-7/LIN28B hormonal axis. circPVT1 sponges miR-145-5p, derepressing Talin1 and supporting EMT-like migration—consistent with the miR-145-5p/Talin1 mechanism [64]. Network discovery analyses nominated dysregulated circRNA–miRNA–mRNA hubs, especially in eutopic endometrium and the EMI [68], although these require luciferase or rescue validation. One study showed cycle-phase circRNA shifts (LH + 2 vs. LH + 7), demonstrating that hsa_circRNA_101280 (derived from SLAIN1) is significantly downregulated in AM during the mid-secretory phase, potentially contributing to impaired endometrial receptivity [54].

### 3.6. EV- and Biofluid-Based Readouts

EV-based studies suggest the feasibility of non-invasive ncRNA biomarker detection. miR-92a-3p in serum and urinary exosomes shows strong discriminatory capacity (AUC 0.9435) [85]. Studies of EV-encapsulated miRNAs with functional readouts (e.g., miR-25-3p, miR-4669) demonstrate effects on EMT and immune remodeling [78,84]. Organoid-derived EVs replicate implantation-related signatures [73]. Vaginal-secreted miRNAs offer feasibility but remain analytically underexplored [76]. However, adherence to MISEV2018 criteria was inconsistent across studies—only five studies fully characterized EVs via TEM, NTA, and marker proteins (CD9/CD63/TSG101) [73,78,84,85,87].

### 3.7. Diagnostic Potential

Across the 41-study corpus, diagnostic readouts are exploratory to discovery-stage and cluster by compartment. In tissue, reciprocal miRNA ratios (e.g., miR-181b/miR-10b) achieved AUC 0.77 (sens 61.3%, spec 72.4%) in eutopic endometrium (ttRT-qPCR), suggesting a low-invasive biopsy concept that requires external validation [58]. A circRNA-based model (tissue hsa_circ_0008959 combined with clinical symptom scores) yielded AUC 0.976 (sens 91.7%, spec 97.2%) in an internal cohort but remains invasive and single-center [77]. In liquid samples, urinary exosomal miR-92a-3p delivered the strongest non-invasive performance (AUC 0.944) and correlated with symptom burden [85]; serum EV miR-4669 and EV miR-25-3p tracked disease biology (M2 → TGF-β1 → EMT) and burden but lack AUC-level diagnostic validation [78,84]. Menstrual-phase stromal EV miRNAs and organoid-EV cargo indicate phase-dependent and receptivity-linked biology relevant for timing-aware diagnostic design [73,87], while post-HIFU vaginal secretion miRNAs demonstrate monitoring feasibility [76]. Diagnostic performance is summarized in Table 2, and targets with direct causal support (L2/L3) prioritized for translation are listed in Table 3 and Table A1.

### 3.8. Therapeutic Targets

The main targetable pathways include epithelial invasion, stromal remodeling, and JZ proliferation. For epithelium, miR-10b directly represses ZEB1/PIK3CA, restoring E-cadherin and curbing AKT-driven invasion [48]; miR-30c-5p → MAPK1 and miR-183 → MMP-9 further nominate MAPK and MMP nodes for intervention [65,68]. In stroma, miR-124-3p → NRP1 restrains EMT-like motility [63], while miR-21 shows compartment-specific effects—pro-decidualization in eutopic stroma versus pro-survival in ectopic ESCs—arguing for spatially targeted designs [57,83]. At the EMI/JZ, let-7a/LIN28B governs estrogen-responsive SMC proliferation with Hippo–YAP as a functional gate; miR-141-3p dampens JAK2/STAT3 in EMI-SMCs [60,62,79]. Among lncRNAs and circRNAs, TUG1/EZH2/TIMP2 stands out by combining human mechanism with in vivo efficacy [56,71]; circPVT1/miR-145/TLN1 and circ_0061140/miR-141-3p/LIN28B provide ceRNA entry points that converge with the miRNA axes [64,69].

Modalities include miRNA mimics/antagomirs (antagomirs are chemically modified antisense oligonucleotides that bind specific miRNAs and block their function) and ASOs/siRNAs targeting lncRNAs/circRNAs. Given the compartmentalized biology, local uterine delivery (IUD-based depots, intrauterine hydrogels, engineered EVs) is the default route. Mechanism → target → approach → evidence mapping is shown in Table 3; prioritization appears in Table A1.

### 3.9. Summary of Robustness and Validation Gaps

Key axes—miR-10b/ZEB1–PIK3CA, miR-30c-5p/MAPK1, miR-145-5p/Talin1, let-7a/LIN28B, miR-21 (context-dependent), and EV miRNAs (miR-25-3p, miR-4669, miR-92a-3p)—show convergent support across compartments [48,57,60,64,67,78,83,84,85], while lncRNA/circRNA programs (HAND2-AS1–HAND2–FGFR, H19/miR-17/TLR4, TUG1/EZH2/TIMP2, circPVT1/miR-145/TLN1, circ_0061140/miR-141-3p/LIN28B) remain mechanistically plausible pending broader validation [56,59,64,69,71,82]. Persistent gaps include limited rescue specificity, sparse compartment-resolved profiling, inconsistent MISEV reporting in EV studies, and the absence of external, prospective diagnostic validation [73,76,87]. Detailed appraisal and implications are discussed in Section 4.6.

## 4. Discussion

“What is not taught is rarely sought.” AM remains diagnostically delayed and therapeutically constrained [1,2]. The biology we teach and test has long prioritized hormones and histology while overlooking post-transcriptional control. NcRNAs—miRNAs, lncRNAs, circRNAs—reframe this gap: they are compact regulators that couple signaling to phenotype, travel in EVs, and persist in biofluids—yet large parts of these ncRNA classes were only recognized in the late 20th century [13,23,27]. Across 41 primary studies included in this systematic review, we observe a recurring architecture in which miRNAs, lncRNAs, and circRNAs converge on four domains that are clinically recognizable: EMT/invasion, estrogen-responsive proliferation in the JZ, stromal remodeling/decidualization, and immune–stromal reprogramming mediated by EVs. This architecture turns a heterogeneous literature into a tractable map for diagnostics and targeted interventions.

### 4.1. Mechanistic Overview Across Four Domains

#### 4.1.1. Epithelium and Invasion

miRNAs that promote epithelial adhesion and limit motility are downregulated in 532 ectopic endometrial glands in adenomyosis (the epithelial compartment of AM). miR-10b directly represses ZEB1 and PIK3CA, increasing E-cadherin and lowering p-AKT [48]; miR-30c-5p decreases MAPK1 activity; and miR-183 limits MMP-9 [70]. Taken together, PI3K/AKT, MAPK, and MMPs appear as tractable levers for invasion control. Methodologically, these signals are supported by luciferase assays and rescue designs in epithelial cells; weaknesses include small single-center cohorts and limited external replication. Cross-disease evidence supports these invasion axes: in breast cancer, miR-10b consistently drives migration and metastasis, underscoring the therapeutic rationale for targeting PI3K/AKT and EMT highlighted here [88].

#### 4.1.2. JZ/EMI Proliferation Under Estrogen

In the JZ smooth-muscle niche, the let-7a/LIN28B axis behaves as a developmental switch: LIN28B up-regulation and let-7a loss bias SMCs toward proliferation, with functional dependence on Hippo–YAP in JZ models [60,61]. These observations triangulate with clinical imaging that localizes disease to the EMI. The main limitation is the scarcity of in vivo confirmation; the strength is consistency across human tissue and primary SMC experiments.

#### 4.1.3. Stroma and Decidualization

Stromal programs are context dependent. miR-21 promotes decidualization in eutopic stroma but maintains survival and suppresses autophagy in ectopic stromal cells via PI3K/AKT/mTOR [57,83]. Additional stromal axes—miR-124-3p → NRP1 and miR-218-5p → LASP1—dampen EMT-like motility and migration [63,72]. The inference is strong at the level of cell-based mechanism with rescue; clinical correlation is emerging but not uniform.

#### 4.1.4. EV-Mediated Immune and Epithelial Remodeling

EV cargo links stromal immunity to epithelial behavior: patient EV miR-25-3p and miR-4669 drive macrophage M2 polarization and TGF-β1–dependent EMT, connecting liquid-phase signatures to tissue remodeling and symptom burden in vivo [78,84]. These studies satisfy key functional criteria but show variable adherence to MISEV2018 reporting, which tempers generalization. A comparable, functional EV program is documented in endometriosis—promoting proliferation, limiting apoptosis, and yielding serum exosomal miRNA signatures with a diagnostic signal—supporting the transferability of EV-based axes across gynecologic disease [89].

### 4.2. Context from Related Conditions

Cross-disease experience suggests a “common trunk” of ncRNA control—proliferation, EMT/migration, immune–inflammatory crosstalk, and matrix/fibrosis—with disease-specific branches. Context dependence of miR-21 and miR-145 is evident in conditions beyond AM: in ovarian cancer tissue, both miRNAs shift across stage and show non-uniform correlations with estrogen and hypoxia/angiogenesis gene sets, reinforcing the hormonal-context hypothesis advanced here [90,91]. Among all conditions, endometriosis shares the most mechanistic overlap with adenomyosis—not only clinically (pain, infertility) but at the molecular level. miR-21, which promotes survival of ectopic stromal cells via PI3K–AKT–mTOR in AM [83], is similarly upregulated in ectopic endometriotic lesions, where it suppresses apoptosis and enhances proliferation [89]. A recent study demonstrated that both miR-21-5p and miR-145-5p are significantly dysregulated in ectopic and eutopic endometrial tissue from endometriosis patients, mirroring expression patterns seen in endometrial cancer. This reinforces their context-dependent, dual-role behavior in estrogen-responsive pathologies [90]. The consistent dysregulation across compartments of miR-145 suggests a fundamental disruption in cytoskeletal regulation, potentially driven by estrogen-dependent promoter activation. This finding converges with recent reports linking miR-145 to Talin1-mediated motility in endometriosis, yet contrasts with its tumor-suppressive role in gynecological cancers—highlighting context-dependent functionality [64,65,92]. Importantly, the correlation between miR-145 levels and symptom severity supports its dual relevance as both a mechanistic driver and a candidate biomarker [65,81]. While current evidence remains confined to single-center cohorts, the stability of exosomal miR-145 in biofluids positions it as a promising potential target for non-invasive monitoring [15,24,89]. Future studies should evaluate whether local restoration of miR-145 expression attenuates epithelial invasion in vivo. For circRNA–miRNA cooperation, multi-omics data in ovarian cancer show that panels combining circular RNAs with miRNAs can outperform CA125/HE4 in small cohorts and map to MAPK/Wnt/ErbB signaling–supporting the rationale for combined signatures in AM [92]. Exosomal communication is a shared feature between endometriosis and AM: both diseases exhibit EV-mediated angiogenesis, macrophage polarization, and EMT; reported overlaps include miR-92a-3p and miR-25-3p where measured [84,85,89]. This suggests a common strategy of immune evasion and stromal priming via EV-mediated signaling. Moreover, reviews in endometrial tumors consistently position circRNAs as stable, clinically promising biomarkers, further indicating that circular-RNA axes are diagnostically actionable within uterine disease [93].

While leiomyomata share anatomical proximity and hormonal dependence, their ncRNA profile differs. In fibroids, H19 overexpression drives ECM accumulation via TGF-β and collagen regulation [20], whereas in adenomyosis, H19 acts primarily through inflammatory derepression (via miR-17 → TLR4/NF-κB) [59]. Furthermore, the differences from leiomyomata are underscored by lncRNA profiles; reviews highlight lncRNA-driven ECM programs with TGF-β/WNT interlocking as central, an ncRNA milieu that diverges from AM [93]. Similarly, in ovarian cancer, H19 acts as an oncogenic lncRNA by sponging miR-17 to enhance NF-κB signaling [33]—mirroring its role in adenomyosis. While in cancer this supports tumor progression, in AM it contributes to chronic inflammation and tissue remodeling. This illustrates how the cellular environment determines the meaning of ncRNA interactions. In contrast, miR-29c, downregulated in fibroids to promote fibrosis, shows no consistent dysregulation in adenomyosis, underscoring distinct stromal phenotypes: fibrotic scarring vs. invasive hyperplasia. However, both conditions show dysregulation of EZH2-mediated chromatin silencing—via lncRNAs like TUG1 in adenomyosis [56] and DALI in fibroids [20]—suggesting that epigenetic control of smooth muscle identity may be a shared vulnerability. The functional role of ncRNAs is profoundly influenced by hormonal milieu, a principle illustrated in PCOS and ovarian cancer. In PCOS, many miRNAs exhibit phase-dependent expression, and their regulatory impact depends on insulin resistance and androgen excess [21]. This reinforces our observation that estrogen modulates key axes in adenomyosis, such as let-7/LIN28B, where estradiol suppresses let-7a to drive JZ-SMC proliferation [60,61] or miR-145, which is transcriptionally activated by ERα and contributes to pro-inflammatory signaling in EMI [81]. These findings support the broader concept that ncRNA function cannot be interpreted in isolation from hormonal context—a caveat essential for experimental design and therapeutic targeting.

### 4.3. Roles of lncRNAs

lncRNAs operate at both chromatin modification and ceRNA competition and connect to endocrine and inflammatory signaling. In particular, lncRNAs in our corpus act either as chromatin-level recruiters (e.g., TUG1 → EZH2 to silence TIMP2), which alter invasion by rewriting local epigenetic states, or as ceRNA scaffolds (e.g., H19 → miR-17 → TLR4; HAND2-AS1 → HAND2/FGFR), which tune signaling thresholds by sponging miRNAs [56,59,82]. For example, HAND2-AS1 enhances HAND2–FGFR signaling, which is vital for endometrial receptivity [82]. H19 acts as a ceRNA for miR-17, releasing suppression of TLR4—a pathway modulated by levonorgestrel therapy [59]. MIR22HG downregulation, with epigenetic control of miR-2861, constrains endometrial-cell proliferation [66]. TUG1 recruits EZH2 to repress TIMP2 and promotes epithelial migration/invasion; an independent study supports the pro-migratory behavior [56,71]. In stroma, MIR503HG silencing accelerates progression via miR-191 and Wnt/β-catenin [75]. There is widespread lncRNA dysregulation in eutopic endometrium and ectopic–eutopic contrasts [50,51]. Cross-entity analysis shows decreases in ENST00000414116 and ENST00000448179 in adenomyosis, but stronger changes in endometriosis/recurrent implantation failure; so patterns are informative for endometrial biology but do not define adenomyosis-specific markers [55]. Because many annotated lncRNAs may reflect transcription at regulatory DNA rather than RNA-encoded functions, prioritizing adenomyosis lncRNAs for diagnostics/therapeutics should weigh RNA-level causality and cellular localization/biogenesis features that affect detectability in biofluids/EVs [45,46].

### 4.4. Roles of circRNAs

circRNAs mostly operate as sponges (e.g., circPVT1 against miR-145; circ_0061140 against miR-141-3p), thereby intersecting the same cytoskeletal and proliferation programs logged by the miRNA work [64,69]. Beyond sponging, circRNAs can, in other systems, bind RNA-binding proteins or influence transcription via nuclear retention [35,36]; in AM, such roles remain inferential but are suggested by network analyses that nominate circRNA–miRNA–mRNA hubs and predict candidate interaction interfaces [68,77]. For example, circ_0061140 enhances LIN28B expression, reinforcing the let-7/LIN28 regulatory loop [69]. Functional annotations also implicate circPVT1 in cytoskeletal remodeling via miR-145 inhibition: circPVT1 sponges miR-145 to derepress Talin1 and enhance migration [64]. Network-based predictions identify additional circRNA–miRNA–mRNA hubs, particularly within eutopic tissue and JZ regions [68,77], although most await experimental validation. Cycle-phase variability further complicates interpretation; studies show that circRNA levels fluctuate significantly between LH + 2 and LH + 7 endometrium [54]. The practical implication is that some mechanisms present multiple therapeutic handles—miRNA replacement, antisense to a circRNA, or silencing of an lncRNA recruiter—supporting combination or sequential designs. As biomarkers, circRNAs’ structural stability and EV-packaging favor biofluid detection, consistent with discovery-stage tissue and liquid signals; their resilience in plasma and exosomes makes them suitable for non-invasive diagnostics. Experience from endometrial and ovarian cancer supports circRNAs—alone or paired with miRNAs—as robust, non-invasive biomarkers, anchoring the mechanisms discussed here within a gynecologic framework [92,93].

### 4.5. Diagnostic Implications

A practical translational precedent already exists in a closely related condition: in endometriosis, a salivary microRNA-based diagnostic (EndoTest^®^) demonstrated excellent performance in a prospective study (sensitivity 96.7%, specificity 100%, AUC 98.3%), with strong external validation (AUC 0.96) [25,26]. This proof-of-concept underscores the feasibility of applying similar miRNA-based strategies to adenomyosis. Converging evidence from oncology strengthens this direction: in colorectal cancer, EV-derived miRNA panels combined with clinical markers and AI-driven modeling achieved stage-spanning performance (AUC 0.99), illustrating how composite signatures and standardized EV workflows can outperform single markers and enable early detection [94]. Likewise, in breast cancer, exosomal miRNAs are highlighted as stable, disease-informative cargo suitable for panel-based liquid biopsy and longitudinal monitoring—an approach directly transferable to benign gynecologic disease where tissue access is limited [95]. More broadly, exosomal ncRNAs are now recognized as active mediators of intercellular crosstalk and phenotype maintenance, reinforcing their pathophysiologic relevance and the value of compartment-aware sampling strategies [96].

However, current diagnostic efforts in AM are still exploratory. A tissue-based qPCR panel of reciprocally dysregulated miRNAs achieved AUC 0.74–0.77 for adenomyosis vs. controls but was only internally evaluated [58]. The best-performing discovery model combined symptom scores with hsa_circ_0008959 and reached AUC 0.976 (Sens 91.7%, Spec 97.2%) internally but requires invasive biopsy and external validation [77]. Liquid candidates are further along conceptually: beyond AM, combined circRNA–miRNA panels show superior discrimination and EV-based miRNA signatures demonstrate diagnostic potential—a trajectory that should be tested systematically in AM [89,92,93]. Urinary exosomal miR-92a-3p delivered AUC 0.9435 and correlated with symptom burden [85]. Serum EV miR-4669 and EV miR-25-3p reflect disease biology (M2 → TGF-β1 → EMT) and burden but lack AUC-level diagnostic validation [78,84]. Menstrual-phase stromal EV miRNAs and organoid-EV cargo argue for timing-aware study designs [73,87]. Post-HIFU vaginal secretion miRNAs show monitoring feasibility [76]. Taken together—and consistent with EV-based diagnostics in oncology—priorities now include composite EV-ncRNA panels, rigorous external validation, and harmonized pre-analytics (EV isolation/normalization) to translate these signals into clinically useful tests [94,95,96].

### 4.6. Therapeutic Targets: Mechanisms and Candidate Interventions

Actionable axes span epithelium, stroma, and the JZ. In epithelium, miR-10b replacement curbs AKT-driven invasion via ZEB1/PIK3CA repression [48]; miR-30c-5p → MAPK1 and miR-183 → MMP-9 nominate MAPK and MMP nodes for intervention [70]. In stroma, miR-124-3p → NRP1 restrains EMT-like motility [63], while miR-21 shows compartment-specific effects—pro-decidualization in eutopic stroma versus pro-survival in ectopic ESCs—arguing for spatially targeted designs [57,83]. At the EMI/JZ, let-7a/LIN28B governs estrogen-responsive SMC proliferation with Hippo–YAP as a functional gate; miR-141-3p dampens JAK2/STAT3 in EMI-SMCs [60,61,79]. Cross-links to mTOR/eIF signaling in benign endometrial disease further support prioritizing targets proximal to PI3K–AKT/mTOR in AM [97]. Among lncRNAs and circRNAs, TUG1/EZH2/TIMP2 stands out by combining human mechanism with in vivo efficacy [56,71]; circPVT1/miR-145/TLN1 and circ_0061140/miR-141-3p/LIN28B provide ceRNA entry points that converge with the miRNA axes [64,69]—a convergence consistent with ceRNA network logic [98].

Building on closely related endometrial cancer (EC) biology, EZH2 emerges as a druggable chromatin effector that interfaces with several of the same cascades implicated in AM. In EC models, miR-137 suppresses EZH2/LSD1, reduces proliferation, and shows in vivo activity, illustrating a miRNA → EZH2 causal route with therapeutic relevance [99].

EC also provides multiple examples of lncRNA–miRNA–EZH2 circuitry (e.g., NEAT1 elevating EZH2 via miRNA suppression), supporting the feasibility of disrupting lncRNA–EZH2 interactions to rebalance estrogen-responsive and inflammatory signaling in endometrium-derived tissues [100].

In parallel, small-molecule EZH2 inhibitors have reached early clinical testing in oncology and demonstrate proof-of-principle target engagement and pathway reversal, suggesting a complementary, drug-class based lever that could be adapted to benign gynecologic indications with local delivery [99].

Modalities include miRNA mimics/antagomirs and ASOs/siRNAs targeting lncRNAs/circRNAs, with local uterine delivery (IUD-based depots, intrauterine hydrogels, or engineered extracellular vesicles) as the default route to maximize on-target exposure and limit systemic effects. Together, these EC-anchored analogies motivate a dual strategy for AM—restoring or inhibiting specific ncRNAs that gate EZH2-linked axes and, in parallel, directly modulating EZH2—both aligned with spatially targeted delivery and rational combinations to address pathway redundancy [99,100].

Modalities include miRNA mimics/antagomirs and ASOs/siRNAs for lncRNAs/circRNAs, with local uterine delivery (IUD-based depots, intrauterine hydrogels, or engineered extracellular vesicles) as the default route to maximize on-target exposure and limit systemic effects. In related translational fields, exosomal ncRNAs function both as actionable cargo and delivery vehicles, and RNA therapeutics increasingly pair with nano-delivery and rational combinations (e.g., pathway or immune-modulatory partners) to overcome biodistribution and off-target barriers—principles that inform AM-focused designs [96,101].

### 4.7. Robustness and Validation Gaps

Signals with convergent support include miR-10b/ZEB1–PIK3CA, miR-30c-5p/MAPK1, miR-145-5p/Talin1, let-7a/LIN28B, compartment-dependent miR-21, and EV miRNAs (miR-25-3p, miR-4669, miR-92a-3p) [48,57,60,65,78,83,84,85]. lncRNA/circRNA programs (HAND2-AS1–HAND2–FGFR, H19/miR-17/TLR4, TUG1/EZH2/TIMP2, circPVT1/miR-145/TLN1, circ_0061140/miR-141-3p/LIN28B) are biologically plausible with preliminary causal support but need broader validation [56,59,64,69,71,82]. Persistent gaps include incomplete rescue specificity, sparse compartment-resolved profiling, inconsistent MISEV reporting in EV studies, and a lack of external, prospective diagnostic validation [73,76,87]. The practical implications include but are not limited to the following: prospective, phase-matched multi-compartment biobanks; blinded validation of liquid candidates (urinary exo-miR-92a-3p; serum EV miR-4669/miR-25-3p); spatial profiling of EMI/JZ to resolve context-dependent axes (miR-21 dichotomy; let-7/LIN28B at JZ); MISEV-complete EV workflows with dose normalization; and local delivery platforms (IUD-based depots, hydrogels, engineered EVs) for uterine targeting.

### 4.8. Strengths and Limitations

The strengths of this first systematic review addressing the role of ncRNAs in AM include, among others, a structured synthesis aligned to compartments (epithelium, stroma, JZ/EMI, EVs), a quality appraisal keyed to design (NOS, SYRCLE, MISEV2018, TRIPOD, omics checklist), a multi-faceted translational perspective, an explicit mechanism/biomarker/intervention crosswalk with evidence tiers (Table 2 and Table 3), as well as a criteria-based prioritization of targets (Table A1) and compartment- and phase-aware data extraction that preserves spatial/temporal context and helps reconcile context-dependent effects (e.g., the miR-21 dichotomy).

The limitations include small single-center cohorts, many relying on overexpression/knockdown without rescue specificity, or reporting discovery-stage omics without false discovery rate control or orthogonal validation. EV papers variably meet MISEV2018 (particle counts, TEM/NTA, positive/negative markers, dose normalization, uptake/rescue assays), which affects transportability of liquid-biopsy claims. Cycle-phase matching and comparator definitions are uneven, confounding tissue studies. These issues constrain meta-analytic synthesis and justify our conservative upgrade/downgrade rules.

## 5. Conclusions

This review provides the first systematic synthesis of ncRNA evidence in adenomyosis, integrating human, experimental, and extracellular-vesicle (EV) studies. Against the backdrop of diagnostic delay and ambiguity in adenomyosis—and the substantial disease burden—the preliminary translational signals summarized here offer cautious hope, provided they are confirmed in prospective, multi-center studies. Current evidence indicates that miRNAs regulate core processes in adenomyosis—epithelial invasion, stromal remodeling, and hormone-responsive proliferation in the junctional zone (JZ). LncRNAs and circRNAs add regulatory layers through chromatin remodeling and competing endogenous RNA (ceRNA) networks. EVs offer a minimally invasive sampling platform that links molecular signatures to immune remodeling and systemic change. From this synthesis, we identify a shortlist of tissue- and EV-supported miRNAs with links to PI3K–AKT/MAPK, JAK–STAT, and Wnt/β-catenin pathways, supporting the feasibility of urine- and serum-based assays for diagnosis and treatment monitoring. Realizing this promise will require prospective, multi-center external validation, harmonized pre-analytics for EV workflows, and—on the interventional side—uterine-local delivery strategies that respect compartmental biology.

## Figures and Tables

**Table 1:** Non-coding RNAs: mechanisms and clinical relevance in AM (descriptive extraction; interpretive synthesis in Section 3.2, Section 3.3, Section 3.4, Section 3.5 and Section 3.6).

Study	RNA Class	Key RNAs	Compartment	Main Findings	Clinical Relevance
Gonzalez, 2009 [47]	miRNA (global)	Global miRNA def (Dicer KO, Amhr2-Cre)	Cell line/primary	Dicer KO → ectopic glands in myometrium, loss of normal glands; aberrant Wnt4/5a/11; systemic E2/P4 unchanged → miRNA–Wnt pathway drives invasion	Causal role: global miRNA loss induces AM-like phenotype; points to miRNA/Wnt-targeted therapies
Guo, 2015 [48]	miRNA	miR-10b ⊣ ZEB1 & PIK3CA → ↑E-cad, ↓p-AKT	Eutopic/ectopic	miR-10b↓ in AM (eutopic < ectopic); OE ↓EEC migration/invasion; directly targets ZEB1 & PIK3CA (luciferase); ↑E-cad, ↓p-AKT; ZEB1/PIK3CA↑ in AM; their KD mimics miR-10b OE	Pathogenesis/target: restore miR-10b or inhibit ZEB1/PIK3CA–AKT to curb invasiveness
Herndon, 2016 [49]	miRNA (microarray)	miR-9-1, -139, -149, -197, -326, -339↑	Eutopic/ectopic	140↑/884↓ genes; above miRNAs↑; dysregulated: EIF2, OXPHOS, ER, mTOR, apoptosis, ECM remodeling	Exploratory pathogenesis: miRNAs link hormone/ECM dysfunction in AM
Jiang, 2016 [50]	lncRNA	165 lncRNAs (6 validated)	Eutopic/ectopic	Discovery: 165 DE lncRNAs (↑48, ↓117); 612 DE mRNAs; qRT-PCR: 3↑/3↓ validated; co-expression: TLN1, MAPK, PI3K–Akt, focal adhesion	Pathogenesis (discovery)
Zhou, 2016 [51]	lncRNA (disco-very)	24 dysregulated lncRNAs; validated: uc004dwe.2↑ (co-exp with NRP2), ENST00000454594, NR_003521	Cell line/primary	388↑/188↓ lncRNAs; 586↑/305↓ mRNAs in ectopic vs. eutopic; enriched: vasculogenesis, cell-matrix adhesion, prolif/EMT, NF-κB/immune	First lncRNA landscape in AM; nominates axes (e.g., uc004dwe.2–NRP2) for future studies
Hu, 2017 [52]	miRNA	miR-17 ⊣ PTEN (direct 3′UTR)	Eutopic/ectopic	AM: miR-17↑, PTEN mRNA/protein↓; miR-17 KD ↑PTEN, ↓viability, ↑apoptosis, ↓CyclinD1/E1; PTEN OE mimics KD; luciferase confirmed miR-17 → PTEN	Pathogenesis/target: miR-17–PTEN axis promotes survival; anti-miR-17 or PTEN-restoration as strategy
Xu, 2018 [53]	lncRNA	Linc-ROR → PI3K–AKT (PTEN↓, p-AKT↑, p-PDK1↑)	Eutopic/ectopic	AM: Linc-ROR↑ (ectopic > eutopic > normal); PTEN↓, AKT↑; Linc-ROR KD ↑PTEN, ↓p-AKT/p-PDK1, ↓prolif; OE opposite; Linc-ROR↑ correlates with diffuse subtype, dysmenorrhea severity	Pathogenesis/target: inhibit Linc-ROR to attenuate PI3K–AKT-driven epithelial prolif
Hu, 2019 [54]	circRNA	hsa_circRNA_101280 (SLAIN1-derived)↓ in AM (LH + 7) vs. Ctrls; predicted sponges: miR-491-5p, -141-5p, -200b-3p, -200c-3p, -429	Cell line/primary	Phase: hsa_circRNA_101280↑ LH + 7 vs. LH + 2; several circRNAs ↓ in AM (LH + 7); hsa_circRNA_101280↓, HOXA10↓, SLAIN1 unchanged; predicted pathways: PI3K–AKT, Wnt, mTOR, Hippo, Hedgehog	Phase-specific receptivity signature; AM-linked ↓hsa_circRNA_101280 may contribute to implantation defects
Li, 2019 [55]	lncRNA	ENST00000433673 → ITGAL → ICAM1 (adhesion)	Cell line/primary	ENST00000433673↓ in AM (and EMs/RIF) vs. normal; predicted ↑ITGAL; associates with ICAM1, EEC adhesion; ITGAL/ICAM1↑ in normal endometrium/EECs	Receptivity axis: ENST00000433673–ITGAL–ICAM1 impaired in AM → implantation defect?
Shi, 2019 [56]	lncRNA	EGR1 → TUG1 → recruits EZH2 ⊣ TIMP2 → ↑epithelial migration/ invasion (prolif unchanged)	Cell line/primary	TUG1↑ in AM epithelia; EGR1 transcriptionally activates TUG1; TUG1 recruits EZH2 → ↑H3K27me3 ⊣ TIMP2 → ↑migration/invasion; TUG1 KD restores TIMP2, ↓invasion	Invasion axis: EGR1–TUG1–EZH2–TIMP2; suggests TUG1/EZH2 inhibition or TIMP2 restoration as therapy
Yan, 2019 [57]	miRNA	miR-21 ⊣ KLF12; modulates NR4A1 (decidualization)	Cell line/primary	AM: miR-21↓; miR-21↑ promotes decidualization (PRL, IGFBP-1, morphology) via ↓KLF12, NR4A1 modulation; inhibition impairs decidualization; KLF12 OE abolishes miR-21 effect	Infertility mechanism: restore miR-21 or target KLF12/NR4A1 to rescue decidualization
Borisov, 2020 [58]	miRNA	miR-10b↓, miR-191↑, miR-200c↓; miR-181b/miR-10b ratio	Eutopic/ectopic	AM vs. Ctrl: miR-10b↓, miR-191↑, miR-200c↓; best ratio miR-181b/miR-10b: AUC 0.77, sens 61.3%, spec 72.4%; other pairs AUC 0.74–0.76	Diagnostic concept: tissue miRNA ratios for low-invasive testing; exploratory
Liang, 2020 [59]	lncRNA/miRNA	H19 → miR-17 ⟂ TLR4 (ceRNA)	Eutopic/ectopic	AM: H19↓, miR-17↑, TLR4↑; LNG or H19 OE ↓miR-17, ↓TLR4, G1 arrest, ↑apoptosis, ↓migration/invasion, ↑E-cad, ↓N-cad/β-cat, ↓MD2/MyD88/NF-κB; miR-17 KD or TLR4 KD mimic effects; luciferase confirmed miR-17 binding to H19 & TLR4	Therapy: LNG ameliorates AM via H19/miR-17/TLR4–NF-κB; targets: ↑H19/ ↓miR-17/↓TLR4
Lin, 2020 [60]	miRNA	let-7a ↔ Lin28B (RBP); Lin28B↑ suppresses let-7a → ↑JZ-SMC prolif	EMI/JZ-SMCs	Lin28B↑ in AM-JZ (mRNA/protein); neg corr with let-7a (r ≈ −0.84); Lin28B KD → let-7a↑, ↓JZSMC prolif; Lin28A no change	JZ growth axis: restore let-7a or inhibit Lin28B to curb JZ-SMC hyperplasia
Huang, 2021 [61]	miRNA	E2 → let-7a/LIN28B axis	EMI/JZ-SMCs	JZ-SMCs: let-7a↓, Lin28B↑; let-7a OE ↓Lin28B, ↓prolif; let-7a inhib ↑Lin28B, ↑prolif; E2 ↓let-7a, ↑Lin28B → estrogen collaborates with let-7a/Lin28B to drive JZ-SMC growth	Hormone–miRNA mechanism: restore let-7a or inhibit Lin28B to curb JZ hyperplasia
Huang, 2021 [62]	miRNA	Let-7a ↔ Hippo--YAP1 axis	JZ-SMC	Let-7a ↓ in AM-JZ-SMCs; let-7a OE ↑ apoptosis, ↓ proliferation via Hippo–YAP1 activation (↑ p-YAP1, ↓ YAP1/TAZ); effect abolished by verteporfin (YAP1 dephosphorylation) → let-7a requires intact Hippo signaling	Therapeutic axis: let-7a restoration + Hippo activators (e.g., verteporfin analogs) to curb JZ-SMC hyperplasia; first demonstration of Hippo-dependency for let-7a in AM.
Huang, 2021 [63]	miRNA	miR-124-3p ⊣ NRP1	Eutopic/ectopic	AM: miR-124-3p↓, NRP1↑; miR-124-3p mimic ↓ESC viability/migration, reverses EMT (↑E-cad, ↓Vim/N-cad/MMP9); inhibitor opposite; NRP1 OE rescues mimic → miR-124-3p ⊣ NRP1 drives EMT/motility	Pathogenesis; therapeutic target
Wang, 2021a [64]	circRNA/miRNA	circPVT1 → miR-145 ⊣ TLN1	Eutopic/ectopic	AM: circPVT1↑, miR-145↓, TLN1↑; circPVT1 sponges miR-145; miR-145 ⊣ TLN1 (luciferase); circPVT1 KD ↓prolif/invasion, OE ↑; miR-145 rescues circPVT1 KD; circPVT1↑ correlates with worse dysmenorrhea, larger uterus	Pathogenesis/target: circPVT1–miR-145–TLN1 axis as therapeutic node
Wang YY, 2021b [65]	miRNA–mRNA axis	miR-145-5p ⊣ Talin1	Cell line/primary	Talin1↑ in AM; miR-145-5p↓, directly binds Talin1 3′UTR; Talin1 OE activates Wnt/β-cat, induces EMT (↓E-cad/cytokeratin; ↑N-cad/vim/Snail/Slug/Twist/ZEB1), ↑migration/invasion; miR-145-5p rescues → ↓Talin1, reverses EMT/motility	Pathogenic axis: miR-145-5p/Talin1 drives epithelial invasion; restore miR-145-5p or inhibit Talin1/Wnt–β-cat
Yu, 2021 [66]	lncRNA/miRNA	MIR22HG → demethylation → ↑miR-2861 ⊣ STAT3/MMP2	Eutopic/ectopic	MIR22HG↓, miR-2861↓ in AM; positively correlated; MIR22HG OE ↓miR-2861 methylation → ↑miR-2861; MIR22HG/miR-2861 ↓STAT3/MMP2, ↓prolif (co-OE strongest)	Pathogenesis/target: boost MIR22HG/miR-2861 or demethylate miR-2861 to suppress STAT3/MMP2
Zhang, 2021 [67]	miRNA	miR-30c-5p ⊣ MAPK1 (direct 3′UTR)	Cell line/primary	miR-30c-5p↓ in AM tissues/epithelia; OE ↓prolif/migration/invasion; KD opposite; MAPK1 OE rescues miR-30c-5p OE; lower miR-30c-5p associates with dysmenorrhea, longer duration, heavier PBAC	Therapeutic axis: restore miR-30c-5p or inhibit MAPK1 to curb epithelial aggressiveness
Guo, 2022 [68]	circRNA	Co-dysregulated: ↑hsa_circ_0002144, _0005806; ↓hsa_circ_0079536, _0024766; ceRNA network → MAPK prominence	EMI/JZ-SMCs	EMI: 760↑/119↓ circRNAs; eutopic: 47↑/17↓; 4 co-dysregulated; ceRNA network (4 circ, 6 miR, 1775 mRNA); MAPK top pathway (also PI3K–AKT, Ras, Hippo)	Atlas: shared circRNA programs across EMI/eutopic drive invasion via MAPK; candidate biomarkers/targets
Li, 2022 [69]	circRNA/ miRNA	circ_0061140 → miR-141-3p → LIN28B	Eutopic/ectopic	circ_0061140↑; cytoplasmic, RNase R-resistant; KD ↓viability/prolif (EdU), ↓migration/invasion (scratch/Transwell), ↑apoptosis, ↓CyclinD1/MMP9; sponges miR-141-3p; miR-141-3p ⊣ LIN28B; LIN28B↑ in AM; anti-miR-141-3p rescues KD; LIN28B OE rescues miR-141-3p → circ_0061140 → miR-141-3p → LIN28B axis	Pathogenesis; therapeutic target (disrupt axis); in vitro only
Wang & Chen, 2022 [70]	miRNA	miR-183 ⊣ MMP-9 (direct 3′UTR)	Cell line/primary	AM: miR-183↓, MMP-9↑; miR-183 OE ↓epithelial viability/migration/invasion; KD ↑; MMP-9 direct target (luciferase); protein ↓with miR-183 mimic	Therapeutic axis: restore miR-183 or inhibit MMP-9 to limit epithelial invasiveness
Yuan, 2022 [71]	lncRNA	TUG1 → binds E2F4 ⊣ KLF5 → ↑EEC prolif/migration/invasion/EMT/angiogenesis; sh-TUG1 reverses	Cell line/primary	TUG1↑ in AM (human/mouse); KD ↓EEC prolif/migration/invasion, reverses EMT (↑E-cad, ↓N-cad), ↓angiogenesis (↓VEGF/CD34); in mice: sh-TUG1 ↓myometrial infiltration/fibrosis, ↓uterine weight/E2; mech: TUG1–E2F4 represses KLF5; KLF5 mod rescues	Therapeutic axis: TUG1–E2F4–KLF5 (in vivo efficacy shown)
Zhang, 2022 [72]	miRNA	miR-218-5p ⊣ LASP1 → ↓Vimentin/EMT → ↓ESC migration	Cell line/primary	miR-218-5p enriched in vascular/myometrium, ↓in AEu/AEc; LASP1 inversely correlated; miR-218-5p OE ↓LASP1, ↓ESC migration, ↓Vimentin, hinders EMT; AM tissues ↑LASP1/Vimentin in epithelium	Therapeutic axis: enhance miR-218-5p or inhibit LASP1 to limit stromal migration at interface
Juárez-Barber, 2023 [73]	miRNA (EV cargo)	Secretory: ↑miR-21-5p, -24-3p, -26a-5p, -92a-3p, -92b-3p, -200c-3p, -423-5p; Gestational: ↑miR-21-5p, -26a-5p, -30a/c-5p, -222-3p, -423-5p	Eutopic/ectopic	AM organoid EVs (100–400 nm): phase-specific miRNA sets (80 secretory, 60 gestational, 54 shared); linked to prolif/invasion/EMT/angiogenesis; PTEN, MDM4, PLAGL2, CELF1↓ → impaired receptivity	Implantation failure hypothesis: endometrial EV miRNAs as biomarkers for pregnancy complications
Tang, 2023 [74]	miRNA	27 ↑miRNAs (e.g., miR-486-5p, -181a-5p, -221-3p, -21-5p, -25-3p, let-7a-3p)	EV/biofluid	Bromocriptine: anti-proliferative/inhibits ESC migration (BrdU/wound/Transwell); local PRL unchanged; 27 ↑miRNAs implicate PI3K–AKT, JAK–STAT, cell cycle, senescence	Therapeutic signal: bromocriptine as candidate therapy via miRNA-linked pathways
Xu, 2023 [75]	lncRNA	MIR503HG ⊣ miR-191 → ↓Wnt/β-cat (↓β-cat), ↑E-cad/↓N-cad → ↓ESC viability/migration/invasion, ↑apoptosis	Cell line/primary	MIR503HG↓ in AM; OE ↓ESC viability/migration/invasion, ↑apoptosis; KD opposite; MIR503HG sponges miR-191 (luciferase, RIP); miR-191 inhibition rescues sh-MIR503HG; KD → EMT/Wnt↑ (↓E-cad, ↑N-cad, ↑β-cat); miR-191 inhib reverses	Therapeutic axis: restore MIR503HG or inhibit miR-191/Wnt–β-catenin to restrain stromal progression
Chen, 2024 [76]	miRNA (biofluid)	Vaginal-secretion miRNA panel shift post-HIFU; AM single case: 41↑/71↓ (miR-7977↓ reported cohort-wide, fibroid focus)	EV/biofluid	Prospective pre/post sampling; small-RNA NGS; diff. expr. analysis; pathway enrichment; no functional assays or external validation	Preclinical HIFU response signature; no AM-specific lead miRNAs identified
Guo, 2024 [77]	circRNA/miRNA	hsa_circ_0008959↓; hsa-miR-124-3p↑	Eutopic/ectopic	AM: circ_0008959↓, SLC15A4↓, miR-124-3p↑; axis: circ_0008959 ⊣ miR-124-3p ⊣ SLC15A4; tissue ROC: AUC 0.826–0.875; circ_0008959 + VAS AUC 0.976 (Se 0.917, Sp 0.972)	Diagnostic concept: high discrimination (tissue + VAS); no external validation; mechanistic inference
Hu, 2024 [78]	miRNA (exoso-mal)	miR-25-3p (EV) → M2 polarization →EEC EMT; PTEN↓/p-AKT↑	EV/biofluid	AM EVs/serum EVs: miR-25-3p↑; EV-treated macrophages → M2 (↑CD163/IL10/ARG1; ↓TNFA/INOS); M2 induce EEC EMT (↓E-cad/CK7; ↑N-cad/Vim); miR-25-3p inhibitors reverse; EECs: PTEN↓, p-AKT↑ after co-culture; baseline EEC migration no difference	Paracrine EV axis: miR-25-3p → M2 → EMT; serum EV miR-25-3p as biomarker hypothesis; no target/RTPCR
Wang, 2024 [79]	miRNA	miR-141-3p ⟂ JAK2/STAT3 (*p*-level)	Cell line/primary	miR-141-3p↓ in AM-EMI SMCs; JAK2/STAT3 (tot/p)↑; miR-141-3p OE ↓prolif, ↑apoptosis; inhibition opposite; WP1066 ↓p-JAK2/p-STAT3, curtailed prolif, abrogated miR-141-3p inhib effects → miR-141-3p restrains EMI SMC growth via JAK2/STAT3 dampening	EMI-focused therapeutic axis: restore miR-141-3p or block JAK2/STAT3 to temper JZ-SMC hyperplasia
Zeng, 2024 [80]	miRNA	miR-21 (ER/E2-regulated)	Eutopic/ectopic	miR-21↑ in ectopic lesions; ER inhibition ↓miR-21 (E2-dep/indep); miR-21 mimic ↑prolif/migration, ↓apoptosis; inhibitor opposite; autophagosome changes with KD	Pathogenesis: ER → miR-21 axis as therapeutic target; no target validation
Zhang, 2024 [81]	miRNA	miR-145 ⊣ CITED2 → ↑NF-κB/HIF-1α → ↑IL-1β/IL-6/VEGF; E2/ERα ↑miR-145; EMT shift (↓E-cad, ↑Vim) → ↑stromal migration	Cell line/primary	miR-145↑ in AM tissues (notably myometrium) and ectopic ESCs; CITED2 direct target (luciferase), ↓with miR-145 OE; CITED2 OE reverses miR-145-induced migration & ↓NF-κB/HIF-1α; E2 via ERα binds pri-miR-145 promoter → ↑miR-145 → ↑VEGF, IL-1β, IL-6, EMT shift	E2–ERα → miR-145 ⊣ CITED2 axis links hormonal signaling to inflammation/angiogenesis/stromal motility; nominate miR-145/CITED2 as target
Zheng, 2024 [82]	lncRNA	HAND2-AS1	Eutopic/ectopic	HAND2/HAND2-AS1↓; FGF9/FGFR–ERK↑; promoter hyper-Me; HAND2-AS1 KD ↓HAND2, ↑FGF9, ↑proliferation/migration → HAND2-AS1 → HAND2/FGF9–ERK axis	Pathogenesis; therapeutic target (restore HAND2-AS1 or inhibit FGFR/ERK)
Jia, 2025 [83]	miRNA	miR-21↑→ PI3K/AKT/mTOR → ↓apoptosis, ↓autophagy, ↑migration (ESC)	Eutopic/ectopic	PI3K/AKT↑ → ↑prolif/↓apoptosis/↑p-mTOR; inhibition reverses; miR-21 mimic ↓apoptosis, ↑migration; inhibitor ↑apoptosis/autophagy; miR-21 enhances 740Y-P pro-mig, reverses LY294002 pro-apoptotic → miR-21 regulates ESC survival/motility via PI3K/AKT/mTOR	Stromal therapeutic axis: miR-21/PI3K/AKT/mTOR
Qiu, 2025 [84]	miRNA (exoso-mal)	exosomal miR-4669 → DUSP6 ⊣ ERK1/2 → M2 macrophage → TGF-β1 →EEC EMT	EV/biofluid	A-eMSC exosomes induce M2; miR-4669↑ in A-exos and serum EVs; correlates with VAS, PBAC, uterine volume; miR-4669 ⊣ DUSP6 → ↑ERK1/2; M2 TGF-β1 mediates EMT; antagomir-4669 limits AM in vivo	Translational: serum exosomal miR-4669 as candidate biomarker; therapeutic axis: miR-4669/DUSP6/ERK → M2 → TGF-β1 → EMT
Shao, 2025 [85]	miRNA (exosomal)	miR-92a-3p↑ (plasma/ lesion exosomes)	EV/biofluid	miR-92a-3p↑ in plasma & lesion exosomes; urinary exosomal miR-92a-3p↑ correlates with uterine vol, VAS, PBAC; AUC (urine) 0.9435; forced miR-92a-3p ↑EEC/ESC migration/invasion, DRG/HUVEC angiogenesis; levels ↓post-hysterectomy	Promising non-invasive biomarker (urinary exosomal); therapeutic candidate (block signaling); target validation needed
Valdés-Bango, 2025 [86]	miRNA (predicted)	IPA-predicted: let-7a-5p, miR-124-3p, miR-16-5p, miR-155-5p	Eutopic/ectopic	Distinct proteomes: external AM → immune/inflammatory; internal AM → migration/apoptosis; IPA predicts phenotype-specific miRNA regulators (internal AM: miRNA-MAPK1/IRF2BP2)	Generates phenotype-specific biomarker/target hypotheses; no ncRNA validation
Zipponi, 2025 [87]	miRNA (exoso-mal)	10 validated: ↑miR-132-5p, -451a, -99a-5p; ↓miR-431-3p, -29c-3p, -337-5p, -144-3p, -590-3p, -1275, -7-5p	Eutopic/ectopic	No diff in exosome number/size; 38 candidates; 10 validated; enriched: PI3K–AKT/mTOR, FoxO, MAPK, integrin, platelet activation → menstruation-specific stromal EV signals linked to survival/migration	Biomarker/target hypothesis: menstrual-phase stromal EV miRNAs

Abbreviations: AM, adenomyosis; EMT, epithelial–mesenchymal transition; JZ, junctional zone; EMI, endometrial–myometrial interface; ESCs, endometrial stromal cells; EECs, endometrial epithelial cells; EV, extracellular vesicle; IHC, immunohistochemistry; ISH, in situ hybridization; qRT-PCR, quantitative reverse-transcription PCR; RIP, RNA immunoprecipitation; ChIP, chromatin immunoprecipitation; EdU, 5-ethinyl-2′-desoxyuridin; AUC, area under the ROC curve; ROC, receiver-operating characteristic. Symbols: → activation/positive regulation; ⊣ inhibition/negative regulation; ↔ bidirectional interaction/crosstalk; ↑ upregulated/increased; ↓ downregulated/decreased; ⟂ independent of/no detectable effect.

**Table 2:** Diagnostic performance of ncRNAs in AM. Evidence levels are defined in Methods; see also Table A1 for prioritization.

Target Category	RNA Axis/Target	Compartment/Sample	Detection Method	Performance Metric (AUC/Sens/Spec)	Evidence Level	Clinical Feasibility	Ref.
Diagnostic (tissue)	Reciprocal miRNA pairs (miR-181b/miR-10b)	Eutopic endometrium (biopsy)	ttRT-qPCR	AUC 0.77 (sens 61.3%, spec 72.4%)	Exploratory	Low-invasive; requires cycle-phase control; needs external cohort	[58]
Diagnostic (tissue)	hsa_circ_0008959 ↓ + VAS	Eutopic endometrium	circRNA-seq + ROC modeling	AUC 0.976 (Se 91.7%, Sp 97.2%)	Discovery (internal)	Highest tissue-based discrimination; requires invasive biopsy; no external validation	[77]
Diagnostic (urine)	Exosomal miR-92a-3p ↑	Urinary exosomes	RT-qPCR	AUC 0.9435	Exploratory	Most promising non-invasive biomarker; correlates with VAS, PBAC, uterine volume; needs target validation	[85]
Diagnostic (serum)	Exosomal miR-4669 ↑	Serum exosomes	miRNA-seq + RT-qPCR	Correlation with VAS/PBAC/uterine vol (no AUC reported)	Mechanistic + correlational	Strong biological plausibility; potential for monitoring disease burden	[84]
Diagnostic (serum)	Exosomal miR-25-3p ↑	Serum exosomes	RT-qPCR	Elevated in AM vs. controls (no AUC)	Mechanistic + correlational	Paracrine EV axis; biomarker hypothesis pending diagnostic validation	[78]
Diagnostic (phase-specific)	Phase-enriched stromal EV miRNAs (e.g., ↑miR-132-5p, ↓miR-431-3p)	Menstrual-phase stromal EVs	Small-RNA seq + qPCR	10 validated miRNAs dysregulated (no AUC)	Biological alignment	Novel menstrual-phase signature; potential for timing-dependent diagnostics	[87]
Diagnostic (receptivity)	ENST00000433673 ↓	Eutopic endometrium	RT-qPCR	Downregulated in AM/RIF vs. normal	Discovery-level	Associated with impaired adhesion (ITGAL/ICAM1); implantation failure biomarker hypothesis	[55]
Diagnostic (receptivity)	hsa_circRNA_101280 ↓ (LH + 7)	Mid-secretory endometrium	circRNA microarray + qPCR	Significant ↓ in AM vs. controls	Discovery-level	Phase-specific signature lost in AM; links to implantation defects	[54]
Monitoring (post-tx)	Vaginal secretion miRNA panel shift	Vaginal secretions post-HIFU	NGS	41↑/71↓ overall; hsa-miR-7977 consistently ↓	Operational feasibility	Proof-of-concept for therapy response monitoring; no AM-specific lead identified	[76]
Feasibility (organoid)	Secretory/gestational EV miRNA cargo	Endometrial organoid-derived EVs	Small-RNA seq	Enriched for pathways linked to implantation failure/pregnancy complications	Biological alignment	Suggests EV miRNA profiles as predictors of reproductive outcomes in AM	[73]
Feasibility (proteomic)	IPA-predicted upstream regulators (e.g., let-7a-5p, miR-124-3p)	Eutopic endometrium	Proteomics + IPA inference	Predicted differential regulation by phenotype (internal vs. external AM)	Hypothesis-generating	No direct ncRNA measurement; generates candidate biomarkers for future validation	[86]

Abbreviations: see Table 1.

**Table 3:** Translational ncRNA-based candidate therapeutic targets in adenomyosis (AM). The mechanism column (evidence from included studies) summarizes experimentally demonstrated effects (directionality, targets, pathways). Evidence levels (L1–L3) are defined in Methods.

Target Category	RNA Axis/Target	Mechanism (in Our Corpus)	Proposed Approach	Evidence Type	Comment	Ref.
Cell proliferation (JZ-SMC)	let-7a/LIN28B	E2-responsive suppression of JZ-SMC proliferation via let-7a targeting LIN28B (r = −0.84)	let-7a mimic or LIN28B ASO	L1 + L2 + L3	Efficacy requires functional Hippo–YAP pathway; combination with verteporfin may enhance response	[60,62]
Cell survival & decidualization	miR-21	Dual role: promotes survival in ectopic ESCs via PI3K/AKT/mTOR; supports decidualization in eutopic stroma via KLF12/NR4A1	AntagomiR-21 (for ectopic ESCs) or miR-21 mimic (for decidualizing stroma)	L1 + L2	Compartment-specific function necessitates spatially targeted delivery	[57,83]
JAK–STAT modulation	miR-141-3p	Suppresses phosphorylation of JAK2/STAT3 in EMI SMC	miR-141-3p mimic	L1 + L2	EMI is a distinct niche—local delivery preferred over systemic	[79]
EMT restraint (stroma)	miR-124-3p	Directly targets NRP1, reversing EMT-like changes in endometrial stromal cells	miR-124-3p mimic	L1 + L2	Promotes stromal quiescence; reduces migratory phenotype	[63]
Migration restraint	miR-218-5p	Inhibits LASP1 expression in endometrial stromal cells, limiting migration at uterine interface	miR-218-5p mimic	L1 + L2	Paracrine signal from vascular endothelium—mimics may restore endogenous barrier	[72]
Epithelial invasion block	miR-10b	Directly targets ZEB1 and PIK3CA, restoring E-cadherin and inhibiting p-AKT-driven invasion	miR-10b mimic	L1 + L2	One of the most robustly validated axes; high priority for preclinical testing	[48]
Inflammation/immune	EV-miR-25-3p	Carried by serum EVs, induces M2 macrophage polarization → TGF-β1 → EEC EMT	Exosome-targeted antagomiR-25-3p	L1 + L2	Novel EV-mediated immune-EMT crosstalk–targetable at source	[78]
Hormonal/inflammatory	H19/miR-17/TLR4	H19 sponges miR-17 → derepression of TLR4 → NF-κB inflammation; LNG suppresses axis	ASO against H19 combined with LNG	L1 + L2	First therapy-linked axis; LNG already clinically used—repurposing potential	[59]
Cytoskeletal/EMT	TUG1/EZH2/TIMP2	TUG1 recruits EZH2 to silence TIMP2 → enhanced epithelial invasion	siRNA against TUG1	L1 + L2 + L3	Strongest preclinical evidence (in vivo efficacy); highest translational readiness	[56,71]
ceRNA/proliferation	circ_0061140 → LIN28B	Sponges miR-141-3p → LIN28B upregulation → proliferation	ASO against circ_0061140	L1 + L2	Aligns with let-7/LIN28B axis–dual targeting potential	[69]
ceRNA/EMT	circPVT1/miR-145/Talin1	circPVT1 sequesters miR-145 → TLN1 upregulation → EMT/migration	ASO against circPVT1	L1 + L2	Convergent with miR-145/Talin1 axis–multiple entry points for intervention	[64]
FGF signaling	HAND2-AS1 → HAND2/FGFR	lncRNA activates HAND2-FGFR–ERK axis → proliferation/migration	ASO against HAND2-AS1	L1 + L2	Links receptivity defect to hyperproliferation—novel hormonal-lncRNA axis	[82]

Abbreviations: see Table 1.

## Data Availability

Dataset available on reasonable request from the authors.

## Supplementary Materials

The following supporting information can be downloaded at: https://www.mdpi.com/article/10.3390/ijms262110713/s1.

## Abbreviations

AEc, adenomyosis ectopic endometrium; AEu, adenomyosis eutopic endometrium; AM, adenomyosis; AUC, area under the curve; ASO(s), antisense oligonucleotide(s); BrdU, 5-bromo-2′-deoxyuridine; CCK-8, Cell Counting Kit-8; CD, cluster of differentiation; ceRNA, competing endogenous RNA; ChIP, chromatin immunoprecipitation; circRNA(s), circular RNA(s); E2, 17β-estradiol; ECM, extracellular matrix; EECs, endometrial epithelial cells; EMI, endometrial–myometrial interface; EMT, epithelial–mesenchymal transition; ESCs, endometrial stromal cells; EVs, extracellular vesicles; FC, fold change; FDR, false discovery rate; FISH, fluorescence in situ hybridization; HIFU, high-intensity focused ultrasound; IHC, immunohistochemistry; IF, immunofluorescence; IPA, Ingenuity Pathway Analysis; IRES, internal ribosome entry site; IUD, intrauterine device; JAK–STAT, Janus kinase/signal transducer and activator of transcription; JZ, junctional zone; JZ-SMCs, junctional zone smooth muscle cells; KD, knockdown; LNG, levonorgestrel; LH, luteinizing hormone; lncRNA(s), long non-coding RNA(s); m6A, N6-methyladenosine; miRNA(s), microRNA(s); MISEV2018, Minimal Information for Studies of Extracellular Vesicles 2018; MRI, magnetic resonance imaging; MTT, 3-(4,5-dimethylthiazol-2-yl)-2,5-diphenyltetrazolium bromide; ncRNA(s), non-coding RNA(s); NGS, next-generation sequencing; NOS, Newcastle–Ottawa Scale; NR, not reported (if used, keep/remove as appropriate); NTA, nanoparticle tracking analysis; OD, optical density; OE, overexpression; PBAC, Pictorial Blood Loss Assessment Chart; PBS, phosphate-buffered saline; PCOS, polycystic ovary syndrome; qRT-PCR, quantitative real-time PCR; RBP, RNA-binding protein; RIF, recurrent implantation failure; RIP, RNA immunoprecipitation; ROC, receiver operating characteristic; Se, sensitivity; sh, short hairpin RNA; si, small interfering RNA; SMC(s), smooth muscle cell(s); Sp, specificity; SYRCLE, Systematic Review Centre for Laboratory animal Experimentation risk-of-bias tool; TEM, transmission electron microscopy; TIAR, tissue injury and repair; TRIPOD, Transparent Reporting of a Multivariable Prediction Model for Individual Prognosis or Diagnosis; TSG101, tumor susceptibility gene 101; TVUS, transvaginal ultrasound; ttRT-qPCR, two-tailed reverse-transcription quantitative PCR; VAS, visual analog scale; WB, Western blot; YAP, Yes-associated protein.

## Appendix A

**Table A1:** Therapeutic strategy matrix according to evidence level from studies included in this systematic review (from “high-priority” to “exploratory”).

Rank	Evidence Level	Target
1. High priority	L3 (in vivo) + L2 (rescue) + L1 (direct target)	TUG1/EZH2/TIMP2, miR-145/Talin1, let-7a/LIN28B
2. Strong candidate	L2 + L1, but no in vivo	miR-141-3p/JAK2/STAT3, miR-218-5p/LASP1
3. Compartment-specific	L1 + L2, but context-dependent	miR-21
4. Biomarker-linked	L1 + clinical correlation, but no functional validation	exo-miR-92a-3p
5. Exploratory	Only bioinformatic or single-case	ENST00000433673

Evidence-level classification: Definitions of L1–L3 appear in Methods (Operational definitions). Table A1 ranks targets by combined evidence (e.g., L1 + L2 + L3). Targets marked L3 (preclinical in vivo) and those with convergent evidence (e.g., miR-145/Talin1, TUG1/EZH2) represent the highest-priority candidates for future development of RNA-based therapies in adenomyosis. For adenomyosis, intrauterine administration (e.g., IUD-based nanoparticles, hydrogel depots) offers superior local bioavailability and reduced off-target effects. Exosomal delivery (e.g., engineered MSC-EVs) is emerging as a promising platform for targeted miRNA replacement or inhibition.
