# Supplementary material for: Non-Coding RNAs (microRNAs, lncRNAs, circRNAs) in Adenomyosis: A Systematic Review of Mechanistic and Translational Evidence
Source: Int J Mol Sci. 2025 Nov 4;26(21):10713. doi: 10.3390/ijms262110713 (PMC12611009; doi:10.3390/ijms262110713)
Supplement: Supplementary file 1 [file ijms-26-10713-s001.zip › Suppl Table S1. Quality and bias.pdf]

Table S1. Risk of bias and quality appraisal.

Study	Title (short)	Study type	Instru-ments applied	MISEV 2018 key items met	Omics QC (norm/FDR/ batch/validati on)	TRIPOD (if model)	In-vitro rigor (controls/ rescue)	Final quality	Notes / limitations
Gonzalez, 2009 [47]	Dicer KO & female tract fertility	Animal model (conditional KO)	SYRCLE	—	—	—	Genetic model; clear phenotype	High	Robust genetic manipulation; adenomyosis-like glands in myometrium; no ncRNA-specific axis
Guo, 2015 [48]	miR-10b ⊣ ZEB1/PIK3CA → ↓p-AKT, ↑E-cad	In vitro mechanistic + tissue	NOS; In-vitro rigor	—	—	—	DLR; rescue	Moderate	Clear axis; discovery cohort modest
Herndon, 2016 [49]	Global transcriptome of eutopic endometrium	Observational omics (microarray)	NOS; Omics QC	—	Normalization + pathway analysis; limited external validation	—	—	Moderate	Small n (3 AM vs 5 Ctrl); discovery-level; qPCR on subset only
Jiang, 2016 [50]	Aberrantly expressed lncRNAs (eutopic endometrium)	Observational omics + qRT-PCR	NOS; Omics QC	—	Normalization reported; limited FDR and orthogonal validation	—	—	Moderate	Microarray with 6 candidates validated; exploratory co-expression
Zhou, 2016 [51]	Ectopic vs eutopic mRNA/lncRNA	Observational omics (paired tissue)	NOS; Omics QC	—	Normalization; bioinformatics; no functional assays	—	—	Moderate	Discovery without mechanistic validation

Hu, 2017 [52]	miR-17 ⊣ PTEN; ↓apoptosis/↑proliferation	In vitro + tissue	NOS; In-vitro rigor	—	—	—	DLR; PTEN overexpression rescue	Moderate	Early single-center; no animal model
Xu, 2018 [53]	Linc-ROR → PI3K-Akt	In vitro + tissue expression	NOS; In-vitro rigor	—	—	—	Basic controls; limited rescue	Low–Moderate	Older methods; limited clinical metadata
Hu, 2019 [54]	CircRNA LH+2 vs LH+7; ↓hsa_circ_101280 in AM	Observational (cycle-phase) + qPCR	NOS; Omics QC	—	Microarray + qPCR; no luciferase	—	—	Moderate	Phase-matched sampling; AM comparison small
Li, 2019 [55]	ENST00000433673 → ITGAL/ICAM1 (receptivity)	Bioinformatics + RT-qPCR (discovery)	NOS	n/a (not an EV study)	No external validation; discovery-level qPCR		n/a (no perturbation experiments)	Moderate	Discovery-level; EEC-enriched expression; no functional assays.
Shi, 2019 [56]	EGR1 → TUG1 ↔ EZH2 ⊣ TIMP2	In vitro + tissue	NOS; In-vitro rigor	—	—	—	ChIP/RIP/pull-down; rescue	Moderate	Strong mechanistic assays; no in vivo
Yan, 2019 [57]	miR-21 → decidualization via ⊣KLF12; NR4A1↑	Human tissue + in vitro decidualization	NOS; In-vitro rigor	—	—	—	Luciferase; rescue; cAMP/MPA protocol	Moderate	Strong in vitro phenotype; no clinical outcomes
Borisov, 2020 [58]	Endometrial miRNA ratios for AM diagnosis	Observational case–control (tissue)	NOS	—	—	— (no multivariable model)	—	Moderate	Single-center; ROC on same cohort; tissue-based; needs noninvasive replication
Liang, 2020 [59]	LNG via H19/miR-17/TLR4	In vitro + tissue expression	NOS; In-vitro rigor	—	—	—	Knockdown/overexpression + DLR	Moderate	Mechanistic axis; clinical translation pending

Lin, 2020 [60]	Lin28B↑; Let-7a↓ in JZ-SMCs	Primary JZ-SMC experiments	NOS; In-vitro rigor	—	—	—	siRNA; proliferation assays	Moderate	Primary cells; no in vivo
Huang, 2021a [61]	E2 ↓let-7a ↑LIN28B → JZ-SMC proliferation	Primary JZ-SMCs + hormone manipulation	NOS; In-vitro rigor checklist				Gain/loss; tissue correlation	Moderate	Overlaps mechanistically mit Lin, 2020; no animal data.
Huang, 2021b [62]	Let-7a ↔ Hippo-YAP1 axis (JZ-SMC)	Primary JZ-SMC experiments	In-vitro rigor	—	—	—	Pathway inhibition (verteporfin)	Moderate	Mechanistic link; no animal model
Huang, 2021c [63]	miR-124-3p ⊣ NRP1; EMT reversal (stroma)	In vitro + tissue expression	NOS; In-vitro rigor checklist				Gain/loss with rescue (NRP1 OE)	Moderate	Single-center; no in-vivo validation.
Wang, 2021a [64]	circPVT1 → miR-145 ⊣ TLN1	In vitro + tissue	NOS; In-vitro rigor	—	—	—	RIP; DLR; rescue	Moderate	Good ceRNA validation; no in vivo
Wang YY, 2021b [65]	Talin1↑ induces EMT; regulated by miR-145-5p	In vitro + tissue	NOS; In-vitro rigor	—	—	—	DLR; EMT markers; rescue	Moderate	Consistent with circPVT1 axis
Yu, 2021 [66]	MIR22HG ↔ miR-2861 → STAT3/MMP2	In vitro + tissue	NOS; In-vitro rigor	—	—	—	Rescue assays; methylation MSP	Moderate	Good mechanistic chain; no in vivo
Zhang, 2021 [67]	miR-30c-5p ⊣ MAPK1	In vitro + tissue	NOS; In-vitro rigor	—	—	—	DLR; phenotypic assays	Moderate	Single-center
Guo, 2022 [68]	Co-dysregulated circRNAs in eutopic/EMI	Observational omics (RNA-seq)	NOS; Omics QC	—	Discovery; ceRNA	—	—	Moderate	Small n; hypothesis-generating

					networks; no functional proof				
Li, 2022 [69]	circ_0061140 ⊣ miR-141-3p → LIN28B	In vitro + tissue	NOS; In-vitro rigor	—	—	—	DLR; RIP; rescue	Moderate	Robust molecular validation; no animal/clinical outcomes
Wang & Chen, 2022 [70]	miR-183 ⊣ MMP-9	In vitro + tissue expression	NOS; In-vitro rigor	—	—	—	DLR; basic controls	Moderate	Limited breadth of validation
Yuan, 2022 [71]	TUG1 → E2F4/KLF5; mouse AM model	In vivo mouse + human tissue + in vitro	SYRCLE; NOS; In-vitro rigor	—	—	—	Rescue; multiple assays	High	Tamoxifen AM model + human concordance strengthen inference
Zhang, 2022 [72]	miR-218-5p ⊣ LASP1; anti-EMT	In vitro + tissue	NOS; In-vitro rigor	—	—	—	DLR; phenotypic assays	Moderate	Microenvironment inference; no in vivo
Juárez-Barber, 2023 [73]	Organoid-EV miRNAs in implantation	Organoid EV + NGS	MISEV2018; Omics QC	Ultracentrifugation; NTA; EV markers	Normalization + FDR; functional enrichment	—	—	High	n=4 organoids; translational relevance
Tang, 2023 [74]	Bromocriptine pre–post + in vitro; miRNA profiling	Pre–post clinical (n=6 AM) + in vitro	NOS; Omics QC; In-vitro rigor	—	Small RNA-seq with enrichment; no external validation	—	Dose–response; replicates	Moderate	Small N; mixed endpoints; mechanistic links suggested

Xu, 2023 [75]	MIR503HG → miR-191 → Wnt/β-catenin	In vitro + tissue	NOS; In-vitro rigor	—	—	—	Rescue assays present	Moderate	Solid cell data; clinical validation limited
Chen, 2024 [76]	Vaginal secretion miRNA pre/post HIFU	Pre–post pilot (biofluid)	NOS; Omics QC	—	NGS; minimal validation	—	—	Low–Moderate	n=8 total; only 1 AM; exploratory
Guo, 2024 [77]	circRNA–miRNA–mRNA network; hsa_circ_0008959 + VAS model	Observational omics + qRT-PCR	NOS; Omics QC; TRIPOD	—	Discovery + partial validation	Partial (development; limited validation)	—	Moderate	Small control n; model not externally validated
Hu, 2024 [78]	EV miR-25-3p → M2 polarization → EMT	Ex vivo EV + in vitro	MISEV2018; In-vitro rigor	Isolation + NTA/TEM + markers; functional chain	—	—	Rescue via miR-25-3p; macrophage polarization	High	Specific pathway; no clinical metrics
Wang, 2024 [79]	miR-141-3p ⊣ JAK2/STAT3 (EMI-SMCs)	Primary EMI-SMC	In-vitro rigor; NOS	—	—	—	Pathway inhibitor (WP1066); rescue	Moderate	Clear downstream pathway; no in vivo
Zeng, 2024 [80]	E2/ER ↔ miR-21; cell phenotypes	Tissue + primary cells	NOS; In-vitro rigor checklist	—	—	—	Phenotypic + ultrastructural readouts	Moderate	Mechanistic link to ER signaling; Chinese-language journal
Zhang, 2024 [81]	miR-145 ⊣ CITED2; E2/ERα↑ miR-145	In vitro + tissue; RNA-seq targets	NOS; In-vitro rigor; Omics QC	—	RNA-seq with validation	—	ChIP-qPCR; rescue	Moderate	Mechanistic + endocrine link; no in vivo

Zheng, 2024 [82]	HAND2-AS1 ↔ HAND2/FGFR; DNA methylation	Human tissue + in vitro silencing	NOS; In-vitro rigor	—	—	—	Silencing with downstream effects	High–Moderate	Multi-modal (IHC/ISH/qPCR/methylation); no animal model
Jia, 2025 [83]	miR-21 → ↓apoptosis/↑migration in EESc via PI3K/AKT/mTOR	In vitro + ex vivo	In-vitro rigor	—	—	—	Pathway activator/inhibitor; phenotypes	Moderate	EESc only; no animal validation
Qiu, 2025 [84]	eMSC-EV miR-4669 → M2 via ¬DUSP6/ ERK; xenograft	Ex vivo EV + in vitro + in vivo xenograft	MISEV2018; SYRCLE; In-vitro rigor	EV characterization; in vivo readout	—	—	Luciferase; rescue; animal validation	High	Strong mechanistic chain with in vivo
Shao, 2025 [85]	EV miR-92a-3p as biomarker; functional effects	Case–control (biofluids/EV) + in vitro	NOS; MISEV2018; In-vitro rigor	Isolation + NTA/TEM + markers; ROC	—	—	Uptake; functional assays	High	Good MISEV compliance; urinary/serum EV metrics
Valdés-Bango, 2025 [86]	Proteomics: internal vs external AM	Observational proteomics	NOS; Omics QC	—	LC-MS DIA; GO/IPA; FDR applied	—	—	High	Well-controlled groups; robust pipeline; ncRNA inferred via upstream analysis
Zipponi, 2025 [87]	Stromal exosome miRNA (menstrual-phase)	Ex vivo EV + small RNA-seq	MISEV2018; Omics QC	Ultracentrifugation + NTA/TEM + markers	TMM/DE + BH-FDR; qPCR validation	—	—	High	Well-characterized EVs; discovery cohort modest

Design-specific instruments were applied as pre-specified in Methods. Where NOS applied, domain subscores are listed when available; otherwise “—”. MISEV2018 items condense isolation, particle and marker characterization.
