# Supplementary material for: Non-Coding RNAs (microRNAs, lncRNAs, circRNAs) in Adenomyosis: A Systematic Review of Mechanistic and Translational Evidence
Source: Int J Mol Sci. 2025 Nov 4;26(21):10713. doi: 10.3390/ijms262110713 (PMC12611009; doi:10.3390/ijms262110713)
Supplement: Supplementary file 1 [file ijms-26-10713-s001.zip › Suppl Table S2 PRISMA_2020_checklist.pdf]

PRISMA 2020 Checklist

Section and Topic	Item #	Checklist item	Location where item is reported
**TITLE**			
Title	1	Identify the report as a systematic review.	Title page: title identifies the article as a systematic review.
**ABSTRACT**			
Abstract	2	See the PRISMA 2020 for Abstracts checklist.	Structured Abstract (Background/Methods/Results/Conclusions).
**INTRODUCTION**			
Rationale	3	Describe the rationale for the review in the context of existing knowledge.	Introduction §1 and §1.2 (rationale in context of existing knowledge).
Objectives	4	Provide an explicit statement of the objective(s) or question(s) the review addresses.	Introduction §1.2, final paragraph (explicit objectives).
**METHODS**			
Eligibility criteria	5	Specify the inclusion and exclusion criteria for the review and how studies were grouped for the syntheses.	Methods §2.1 (Eligibility criteria; grouping for syntheses).
Information sources	6	Specify all databases, registers, websites, organisations, reference lists and other sources searched or consulted to identify studies. Specify the date when each source was last searched or consulted.	Methods §2.2 (Information sources & search strategy; last search: 10 Aug 2025).
Search strategy	7	Present the full search strategies for all databases, registers and websites, including any filters and limits used.	Methods §2.2 (Full database/web search strings, filters, limits).
Selection process	8	Specify the methods used to decide whether a study met the inclusion criteria of the review, including how many reviewers screened each record and each report retrieved, whether they worked independently, and if applicable, details of automation tools used in the process.	Methods §2.3 (Selection process; two independent reviewers; no automation).
Data collection process	9	Specify the methods used to collect data from reports, including how many reviewers collected data from each report, whether they worked independently, any processes for obtaining or confirming data from study investigators, and if applicable, details of automation tools used in the process.	Methods §2.4 (Data collection: three independent reviewers; standardized extraction).
Data items	10a	List and define all outcomes for which data were sought. Specify whether all results that were compatible with each outcome domain in each study were sought (e.g. for all measures, time points, analyses), and if not, the methods used to decide which results to collect.	Methods §2.4 (Outcomes sought: functional readouts, diagnostic metrics, etc.).
	10b	List and define all other variables for which data were sought (e.g. participant and intervention characteristics, funding sources). Describe any assumptions made about any missing or unclear information.	Methods §2.4 (Other variables: compartments, cell types, techniques, pathways, confounders).
Study risk of bias assessment	11	Specify the methods used to assess risk of bias in the included studies, including details of the tool(s) used, how many reviewers assessed each study and whether they worked independently, and if applicable, details of automation tools used in the process.	Methods §2.5 (Risk of bias/quality tools: NOS, SYRCLE, MISEV2018, TRIPOD, omics

Section and Topic	Item #	Checklist item	Location where item is reported
			checklist; independent assessment).
Effect measures	12	Specify for each outcome the effect measure(s) (e.g. risk ratio, mean difference) used in the synthesis or presentation of results.	Methods §2.6 (Effect measures & synthesis methods: AUC/sensitivity/specificity; no meta-analysis).
Synthesis methods	13a	Describe the processes used to decide which studies were eligible for each synthesis (e.g. tabulating the study intervention characteristics and comparing against the planned groups for each synthesis (item #5)).	Methods §2.4 & §2.6 (How studies were assigned to narrative syntheses by RNA class).
	13b	Describe any methods required to prepare the data for presentation or synthesis, such as handling of missing summary statistics, or data conversions.	Methods §2.6 (No data conversions required; heterogeneous endpoints → narrative-only).
	13c	Describe any methods used to tabulate or visually display results of individual studies and syntheses.	Methods §2.6 + Tables 1–2; Supplement Tables S1–S2 (tabulation/visual display).
	13d	Describe any methods used to synthesize results and provide a rationale for the choice(s). If meta-analysis was performed, describe the model(s), method(s) to identify the presence and extent of statistical heterogeneity, and software package(s) used.	Methods §2.6 (Rationale for narrative synthesis; justification against meta-analysis due to heterogeneity).
	13e	Describe any methods used to explore possible causes of heterogeneity among study results (e.g. subgroup analysis, meta-regression).	Methods §2.6 & Discussion (heterogeneity described; no formal subgroup analyses).
	13f	Describe any sensitivity analyses conducted to assess robustness of the synthesized results.	Not applicable (no meta-analysis; no sensitivity analyses).
Reporting bias assessment	14	Describe any methods used to assess risk of bias due to missing results in a synthesis (arising from reporting biases).	Not applicable (no statistical syntheses → no formal reporting-bias assessment).
Certainty assessment	15	Describe any methods used to assess certainty (or confidence) in the body of evidence for an outcome.	Methods §2.5 & Appendix A (no GRADE; conservative down-/upgrade rules; evidence tiers L1–L3).
**RESULTS**			
Study selection	16a	Describe the results of the search and selection process, from the number of records identified in the search to the number of studies included in the review, ideally using a flow diagram.	Methods §2.3 (numbers) + Supplement Figure S1 (PRISMA flow diagram).
	16b	Cite studies that might appear to meet the inclusion criteria, but which were excluded, and explain why they were excluded.	Supplement Figure S1 (full-text exclusions with reasons).
Study	17	Cite each included study and present its characteristics.	Results §3.1 + Table 1 (study characteristics).

PRISMA

PRISMA 2020 Checklist

Section and Topic	Item #	Checklist item	Location where item is reported
characteristics			
Risk of bias in studies	18	Present assessments of risk of bias for each included study.	Supplement Table S1 (risk of bias per study).
Results of individual studies	19	For all outcomes, present, for each study: (a) summary statistics for each group (where appropriate) and (b) an effect estimate and its precision (e.g. confidence/credible interval), ideally using structured tables or plots.	Table 1 and Results §3. (individual study results summarized).
Results of syntheses	20a	For each synthesis, briefly summarise the characteristics and risk of bias among contributing studies.	Results §3.2–§3.8 (synthesis summaries including RoB context).
	20b	Present results of all statistical syntheses conducted. If meta-analysis was done, present for each the summary estimate and its precision (e.g. confidence/credible interval) and measures of statistical heterogeneity. If comparing groups, describe the direction of the effect.	Not applicable (no meta-analysis).
	20c	Present results of all investigations of possible causes of heterogeneity among study results.	Not applicable (no formal heterogeneity analyses); heterogeneity described narratively (Methods §2.6; Discussion).
	20d	Present results of all sensitivity analyses conducted to assess the robustness of the synthesized results.	Not applicable (no sensitivity analyses).
Reporting biases	21	Present assessments of risk of bias due to missing results (arising from reporting biases) for each synthesis assessed.	Not applicable (no statistical syntheses).
Certainty of evidence	22	Present assessments of certainty (or confidence) in the body of evidence for each outcome assessed.	Appendix A (evidence certainty tiers) + Methods §2.5 (rating rules).
**DISCUSSION**			
Discussion	23a	Provide a general interpretation of the results in the context of other evidence.	Discussion (interpretation in context) + Conclusions (Section 5).
	23b	Discuss any limitations of the evidence included in the review.	Discussion (limitations of the evidence).
	23c	Discuss any limitations of the review processes used.	Methods §2.7 (not registered; internal protocol) + Discussion (process limitations).
	23d	Discuss implications of the results for practice, policy, and future research.	Discussion & Conclusions (implications for practice/policy/future research).
**OTHER INFORMATION**			
Registration and protocol	24a	Provide registration information for the review, including register name and registration number, or state that the review was not registered.	Methods §2.7 (review not registered; no PROSPERO ID).
	24b	Indicate where the review protocol can be accessed, or state that a protocol was not prepared.	Methods §2.7 (internal protocol available on request).

PRISMA

PRISMA 2020 Checklist

Section and Topic	Item #	Checklist item	Location where item is reported
	24c	Describe and explain any amendments to information provided at registration or in the protocol.	Not applicable (no protocol amendments reported).
Support	25	Describe sources of financial or non-financial support for the review, and the role of the funders or sponsors in the review.	Funding: no external funding.
Competing interests	26	Declare any competing interests of review authors.	Conflicts of Interest: authors declare no competing interests.
Availability of data, code and other materials	27	Report which of the following are publicly available and where they can be found: template data collection forms; data extracted from included studies; data used for all analyses; analytic code; any other materials used in the review.	Data Availability Statement: data/materials available on request.

From: Page MJ, McKenzie JE, Bossuyt PM, Boutron I, Hoffmann TC, Mulrow CD, et al. The PRISMA 2020 statement: an updated guideline for reporting systematic reviews. BMJ 2021;372:n71. doi: 10.1136/bmj.n71.
This work is licensed under CC BY 4.0. To view a copy of this license, visit https://creativecommons.org/licenses/by/4.0/
