# Supplementary material for: Non-Coding RNAs (microRNAs, lncRNAs, circRNAs) in Adenomyosis: A Systematic Review of Mechanistic and Translational Evidence
Source: Int J Mol Sci. 2025 Nov 4;26(21):10713. doi: 10.3390/ijms262110713 (PMC12611009; doi:10.3390/ijms262110713)
Supplement: Supplementary file 1 [file ijms-26-10713-s001.zip › Suppl Table S3. Study Design and Methodological Details.pdf]

**Supplementary Table S3.** Study Design and Methodological Details of ncRNA Investigations in Adenomyosis

| Study | Key ncRNA Axis | Sample/Compartment | Design and Methods |
|---|---|---|---|
| Gonzalez /Behringer, 2009 | Global miRNA deficiency (Dicer KO, Amhr2-Cre) | Mouse uterus (Amhr2-Cre;Dicer^fx/fx vs Dicer^fx/fx controls); Müllerian mesenchyme-specific KO | Histology + E-cadherin IF for ectopic glands; in situ hybridization (Wnt4/5a/11); serum E2/P4 quantification; causal role established via conditional knockout. |
| Guo, 2015 | miR-10b ⊣ ZEB1 & PIK3CA → ↑E-cad, ↓p-AKT | Eutopic/ectopic tissue: AM n=22 (paired) vs Ctrl n=13; primary EECs | MiRNA microarray → qRT-PCR; miR-10b mimic/anti-miR; Transwell migration/invasion; dual-luciferase (ZEB1/PIK3CA 3′UTRs); IHC/WB; shRNA knockdown of ZEB1/PIK3CA. |
| Herndon, 2016 | Global transcriptome (miRNA-associated pathways) | Eutopic endometrium: AM n=3 vs Ctrl n=5 (hysterectomy/biopsy); free of confounders | Agilent Human Gene 1.0 ST microarray; RMA normalization; BH-FDR; PCA/clustering; IPA pathway analysis; qRT-PCR validation of coding genes (no miRNA data reported). |
| Jiang, 2016 | 165 lncRNAs (discovery set; 6 validated) | Eutopic endometrium (proliferative phase); AM n=4 vs Ctrl n=4 (microarray); validation: AM n=20 vs Ctrl n=16 (qRT-PCR) | Observational case–control: discovery via microarray (n=8 total), followed by qRT-PCR validation of 6 lncRNAs; co-expression network analysis of DE mRNAs. |
| Zhou, 2016 | Discovery: 388↑/188↓ lncRNAs; validated: uc004dwe.2↑, ENST00000454594, NR_003521 | Paired ectopic vs eutopic tissue (AM, n=3 pairs microarray; n=3 additional qRT-PCR) | lncRNA+mRNA microarray (Agilent); qRT-PCR validation (3 lncRNAs, 3 mRNAs); GO/KEGG enrichment; lncRNA–mRNA co-expression network. |
| Hu, 2017 | miR-17 ⊣ PTEN (direct targeting) | Eutopic tissue: AM n=45 vs Ctrl n=32; primary ESCs | Case–Ctrl qRT-PCR/Western; antagomiR-17 knockdown; PTEN OE; MTT viability; flow cytometry apoptosis; dual-luciferase (PTEN 3′UTR). |
| Xu, 2018 | Linc-ROR → PI3K–AKT (PTEN↓, p-AKT↑) | Ectopic/eutopic tissue: AM n=40 vs Ctrl n=40; primary epithelial cells | Case–Ctrl qRT-PCR/Western; si-Linc-ROR/OE; CCK-8 proliferation; correlation with AM subtype (diffuse) and symptom severity. |
| Hu, 2019 | circRNA: hsa_circ_101280↓ in AM (LH+7); predicted sponges: miR-491-5p, -141-5p, -200b-3p, -200c-3p, -429 | Endometrium: LH+2 (n=3/5) vs LH+7 (n=3/5); AM LH+7 (n=11) vs Ctrl (n=11); primary EECs/ESCs | circRNA microarray; qRT-PCR validation; RNase R resistance + back-splice confirmation; cell fractionation; in silico target prediction (TargetScan/miRDB); GO/KEGG; no functional assays. |
| Li, 2019 | ENST00000433673 → ITGAL → ICAM1 (adhesion) | Endometrial tissues: AM vs normal/endometriosis/RIF; EEC-enriched | lncRNA qRT-PCR; bioinformatic target prediction (ITGAL/ICAM1); expression analysis in tissues and EECs; no functional knockdown/overexpression. |

| | | | |
|---|---|---|---|
| Shi, 2019 | EGR1 → TUG1 → recruits EZH2 ⊣ TIMP2 → ↑migration | Eutopic/ectopic tissue: AM n≈30 pairs; primary EECs | qRT-PCR/IHC/IF (TUG1, EGR1); si-EGR1/si-TUG1; Transwell migration/invasion; luciferase (EGR1 → TUG1 promoter); RIP (TUG1–EZH2); ChIP (EZH2/H3K27me3 at TIMP2 promoter). |
| Yan, 2019 | miR-21 ⊣ KLF12 → modulates NR4A1 (decidualization) | Eutopic endometrium (AM vs fertile controls); primary hESCs | RT-qPCR (miR-21); in vitro decidualization (8Br-cAMP+MPA); miR-21 mimic/inhibitor; luciferase (KLF12 3′UTR); Western/qPCR; KLF12 OE rescue. |
| Borisov, 2020 | miR-10b↓, miR-191↑, miR-200c↓; miR-181b/miR-10b ratio | Eutopic endometrium (proliferative); Pipelle biopsies; discovery: AM n=10 vs Ctrl n=10; validation: AM n=33 vs Ctrl n=30 | Two-step case–control: (i) pooled screening (miRCURY panel, 170 miRNAs); (ii) individual ttRT-qPCR validation (global Ct + U6 normalization); ROC analysis of miRNA ratios. |
| Liang, 2020 | H19 → miR-17 ⊥ TLR4 (ceRNA) | Eutopic tissue: AM n=71 vs Ctrl n=54; primary ESCs | Case–Ctrl qRT-PCR/WB; levonorgestrel treatment; gain/loss of H19/miR-17/TLR4; dual-luciferase (miR-17 binding to H19/TLR4); migration/invasion, cell-cycle, apoptosis; NF-κB cytokines (TNF-α, IL-6). |
| Lin, 2020 | LIN28B → ↓let-7a (inverse axis) | JZ tissue: AM n=30 vs Ctrl n=30; primary JZ-SMCs (AM n=8 vs Ctrl n=8) | IHC/WB/RT-qPCR; Pearson correlation (let-7a vs Lin28B); si-Lin28B knockdown; CCK-8 proliferation. |
| Huang, 2021 | miR-124-3p ⊣ NRP1 | Eutopic tissue: AM n=20 vs fibroid controls n=20; primary ESCs | Case–Ctrl qRT-PCR + dual-luciferase (NRP1 3′UTR); miR-124-3p mimic/inhibitor in ESCs; CCK-8, wound-healing, Western; rescue with NRP1 overexpression. |
| Huang, 2021 | E2 → let-7a/LIN28B axis | JZ-SMCs: tissue (AM n=20 vs Ctrl n=20); primary JZ-SMCs (AM n=8 vs Ctrl n=8) | Tissue RT-qPCR (let-7a); Western (Lin28B); lentiviral let-7a OE/inhibition; 17β-estradiol exposure (10 nM); CCK-8 proliferation assays. |
| Huang, 2021 | let-7a ↔ Hippo–YAP1/TAZ — let-7a ↑ → p-YAP1 ↑ → ↓prolif, ↑apoptosis (JZ-SMC) | JZ-SMCs: AM n=25 vs Ctrl n=27 (hysterectomy tissue) | RT-qPCR/Western (let-7a, YAP1/TAZ, p-YAP1); lentiviral let-7a OE; CCK-8; flow cytometry apoptosis; verteporfin to block Hippo–YAP signaling. |
| Wang YY, 2021 | miR-145-5p ⊣ TLN1 → Wnt/β-catenin → EMT | Eutopic/ectopic tissue: AM n=45 vs Ctrl n=40; primary EECs | Case–Ctrl qRT-PCR/IHC; miR-145-5p mimic/inhibitor; TLN1 OE/KD; dual-luciferase (TLN1 3′UTR); EMT marker profiling; migration/invasion; Wnt/β-catenin pathway readouts. |
| Wang, 2021 | circPVT1 → miR-145 ⊣ TLN1 | Eutopic/ectopic tissue: AM n=45 vs Ctrl n=40; primary EECs/ESCs | Case–Ctrl qRT-PCR + IHC/Western; subcellular fractionation; RNase R resistance; dual-luciferase + AGO2-RIP; gain/loss-of-function + rescue; CCK-8, colony |

| | | | |
|---|---|---|---|
| | | | formation, Transwell; correlation with VAS, PBAC, uterine volume. |
| Yu, 2021 | MIR22HG → demethylation → ↑miR-2861 ⊣ STAT3/MMP2 | Eutopic tissue: AM n=45 vs Ctrl n=45; primary cells | Case–Ctrl qRT-PCR; MSP for miR-2861 methylation; MIR22HG OE + miR-2861 mimic; dual-luciferase (STAT3/MMP2 targets); Western; CCK-8. |
| Zhang, 2021 | miR-30c-5p ⊣ MAPK1 (direct 3′UTR) | Eutopic/ectopic tissue: AM n=23 vs Ctrl n=20; primary epithelial cells | Case–Ctrl qRT-PCR; mimic/inhibitor; CCK-8, scratch, Transwell; WB (MAPK1); dual-luciferase (MAPK1 3′UTR); rescue with MAPK1 OE. |
| Guo, 2022 | Co-dysregulated circRNAs: ↑hsa_circ_0002144, _0005806; ↓hsa_circ_0079536, _0024766; ceRNA → MAPK | Eutopic endometrium and EMI (paired tissues, AM n=5 vs Ctrl n=3) | Ribominus + RNase R-enriched circRNA-seq (MGISEQ-2000); DE analysis (DESeq2); ceRNA network (miRanda/circBank/TargetScan/starBase); GO/KEGG enrichment; no qPCR/validation. |
| Li, 2022 | circ_0061140 → miR-141-3p → LIN28B | Eutopic tissue: AM n=27 vs fibroid controls n=15 (proliferative); primary EECs | Case–Ctrl qRT-PCR + RNase R resistance assay; subcellular localization; si-circ_0061140 / miR-141-3p mimic/inhibitor / LIN28B OE/KD; dual-luciferase + RIP; rescue experiments. |
| Wang & Chen, 2022 | miR-183 ⊣ MMP-9 (direct 3′UTR) | Eutopic tissue: AM n=30 vs Ctrl n=30; primary epithelial cells | qRT-PCR (miR-183); IHC/WB (MMP-9); mimic/inhibitor; CCK-8, scratch, Transwell; dual-luciferase (MMP-9 3′UTR). |
| Yuan, 2022 | TUG1 → E2F4 ⊣ KLF5 → ↑prolif/migration/EMT/angiogenesis | Human tissue: AM n=40 vs Ctrl n=40; tamoxifen-induced mouse AM model (sh-TUG1 intrauterine) | Case–Ctrl qRT-PCR; sh-TUG1 in EECs: CCK-8, colony, scratch, Transwell, IF (EMT/angiogenesis); RIP/RNA pull-down (TUG1–E2F4); KLF5 rescue; in vivo: H&E/Masson, uterine weight, serum E2. |
| Zhang, 2022 | miR-218-5p ⊣ LASP1 → ↓Vimentin/EMT → ↓ESC migration | Endometrial tissue: CEu n=17, AEu n=16, AEc n=16; primary ESCs (AM ectopic, Ems, ThESC) | RT-qPCR/FISH/IF (miR-218-5p, LASP1, Vimentin); mimic/inhibitor; Transwell; EdU; dual-luciferase (LASP1 3′UTR). |
| Juárez-Barber, 2023 | Phase-enriched EV miRNAs (secretory/gestational) | AM organoids (n=4); differentiated to secretory/gestational phases; validation cohort (AM n=4 vs Ctrl n=4) | EV isolation (ultracentrifugation); NTA/TEM/Western (TSG101/CD9/CD81); small-RNA seq; target prediction (miRNet); GO/Pathway enrichment; qRT-PCR validation of PTEN, MDM4, PLAGL2, CELF1 downregulation. |
| Tang, 2023 | 27 ↑miRNAs post-bromocriptine (e.g., miR-486-5p, -181a-5p, -221-3p) | Paired eutopic endometrium (baseline vs 6mo bromocriptine); n=6 pre, n=4 post; primary ESCs from same patients | Self-controlled pre/post design: IHC (Ki67), RT-qPCR/ELISA (PRL); in vitro ESC assays (BrdU, wound-healing, Transwell); small-RNA seq (DESeq, FDR<0.05, \|FC\|>2); KEGG enrichment (GEO GSE207522). |
| Xu, 2023 | MIR503HG ⊣ miR-191 → ↓Wnt/β-catenin | Eutopic tissue: AM n=30 vs Ctrl n=30; primary ESCs (vimentin+/cytokeratin−) | qRT-PCR; MIR503HG OE/sh; miR-191 mimic/inhibitor; dual-luciferase + Ago2-RIP; CCK-8, Transwell, apoptosis (flow); WB (E-cad/N-cad/β-cat/caspase-3). |

| | | | |
|---|---|---|---|
| Chen, 2024 | Multiple (41↑/71↓ miRNAs post-HIFU) | Vaginal secretions: AM n=1 + fibroids n=7 (pre/post HIFU) | Prospective pre/post sampling; small-RNA NGS; differential expression analysis; pathway enrichment; no functional validation or AM-specific lead miRNAs identified. |
| Guo, 2024 | circ_0008959 ⊣ miR-124-3p ⊣ SLC15A4 | Eutopic tissue: discovery (AM n=12 vs Ctrl n=3); validation (AM n=36 vs Ctrl n=36); VAS integration | High-throughput circRNA-seq; ceRNA network construction (circRNA–miRNA–mRNA); clinical correlation with VAS score; exploratory diagnostic model (circ_0008959 + VAS). |
| Hu, 2024 | miR-25-3p (EV) → M2 → EEC EMT; PTEN↓/p-AKT↑ | EVs from eutopic cell supernatants and serum: AM n=11 vs Ctrl n=10; THP-1 macrophages, EECs, Ishikawa cells | EV isolation (Exo-spin); TEM/NTA/markers; qRT-PCR (miR-25-3p); macrophage transfection (mimic/inhibitor); co-culture; EMT markers (E-cad/Vim); WB (PTEN/p-AKT); migration assays. |
| Wang, 2024 | miR-141-3p ⊥ JAK2/STAT3 (p-level) | Primary EMI SMCs: AM n=25 vs Ctrl n=20 (ex vivo culture) | RT-qPCR/Western (miR-141-3p, JAK2/STAT3, p-JAK2/p-STAT3); mimic/inhibitor; CCK-8; Annexin-V/PI apoptosis; WP1066 (JAK2/STAT3 inhibitor) rescue. |
| Zeng, 2024 | miR-21 (ER/E2-regulated) | Ectopic lesion vs myometrium (n=12/12); eutopic vs normal endometrium (n=12/12); primary lesion cells (n=4 lines) | RT-qPCR (miR-21); ER inhibition (ICI182780) ± E2; miR-21 mimic/inhibitor; MTT, scratch, apoptosis (flow), TEM ultrastructure. |
| Zhang et al (2024, RBMO), 2024 | E2 → miR-145 → CITED2 → ↑NF-κB/HIF-1α → ↑IL-1β/IL-6/VEGF | Ectopic/eutopic tissue: AM n=28 vs non-AM n=14; primary ESCs (ThESC line) | FISH (miR-145 localization); RT-qPCR/WB; RNA-seq after miR-145 OE; dual-luciferase (CITED2 3′UTR); transwell migration ± CITED2 rescue; ChIP-qPCR (ERα binding to pri-miR-145); E2 stimulation. |
| Zheng, 2024 | HAND2-AS1 ↔ HAND2–FGFR axis | Normal endometrium; paired eutopic/ectopic tissue (AM n=20); ESCs (in vitro KD) | Tissue expression (IHC/ISH/qRT-PCR); promoter/exon methylation (bisulfite sequencing); HAND2-AS1 silencing in ESCs to assess HAND2/FGF9 regulation and cell behavior (proliferation/migration). |
| Jia, 2025 | miR-21 → PI3K/AKT/mTOR → ↓apoptosis/↑migration (EESc) | Ectopic ESCs from AM lesions (n=4 patient cultures) | Primary EESc treated with PI3K/AKT activator (740 Y-P) or inhibitor (LY294002); miR-21 mimic/inhibitor; MTT, Annexin V/7-AAD, scratch, WB (mTOR/p-mTOR), TEM autophagy, qRT-PCR. |
| Qiu, 2025 | exosomal miR-4669 → DUSP6 ⊣ ERK1/2 → M2 → TGF-β1 → EMT | A-eMSCs vs N-eMSCs (n=3/3); serum EVs: AM n=36 vs Ctrl n=36; THP-1 macrophages; Ishikawa cells; AM xenograft mice (n=6/group) | EV isolation (ultracentrifugation); TEM/NTA/markers (CD9/CD63/TSG101); EV-miRNA-seq; qRT-PCR; macrophage polarization (CD163/IL10); TGF-β1 |

| | | | |
|---|---|---|---|
| | | | neutralization; dual-luciferase (DUSP6); ERK modulation (PD0325901); antagomir-4669 in vivo. |
| Shao, 2025 | miR-92a-3p↑ in exosomes | Plasma, lesion supernatant, urinary EVs; AM n=45–41 vs Ctrl n=21–16; recipient cells: EEC, ESC, DRG, HUVECs | EV isolation (ultracentrifugation); TEM/NTA/markers; miRNA profiling + qRT-PCR; exosome uptake imaging; mimic/inhibitor transfection; migration/invasion/proliferation/tube formation; urine ROC; pre/post hysterectomy validation. |
| Valdés-Bango, 2025 | IPA-predicted regulators: let-7a-5p, miR-124-3p, miR-16-5p, miR-155-5p | Eutopic tissue: internal AM n=9, external AM n=9, controls n=18 (proliferative) | Cross-sectional DIA proteomics; Ingenuity Pathway Analysis (IPA) for upstream regulator inference; miRNA predictions derived from proteomic dysregulation patterns; no direct ncRNA measurement. |
| Zipponi, 2025 | 10 validated EV miRNAs (e.g., ↑miR-132-5p, ↓miR-431-3p) | AM-derived stromal EVs (n=11 vs Ctrl n=11); qPCR validation (n=4/4, menstrual phase) | EV isolation (differential ultracentrifugation); NTA/TEM/flow cytometry (CD9/CD81); small-RNA seq (QIAseq, UMIs); DE analysis (∣log2FC∣>2); GO/KEGG/Reactome enrichment; qPCR validation. |

**Table S3-bis:** Abbreviations for Table S3 listed alphabetically; definitions reflect usage in Table S3.

| **Abbreviation** | **Definition** |
|---|---|
| 3′UTR | 3′ untranslated region |
| A-eMSCs | Adenomyosis-derived endometrial mesenchymal stem cells |
| AEc | Adenomyosis ectopic endometrium |
| AEu | Adenomyosis eutopic endometrium |
| AGO2-RIP | Argonaute-2 RNA immunoprecipitation |
| AKT / p-AKT | Protein kinase B / phosphorylated AKT |
| AM | Adenomyosis |
| Amhr2-Cre | Anti-Müllerian hormone receptor type 2-Cre driver |
| AUC / ROC | Area under the ROC curve / Receiver operating characteristic |
| BH-FDR | Benjamini–Hochberg false discovery rate |
| BrdU | Bromodeoxyuridine (proliferation assay) |
| CCK-8 | Cell Counting Kit-8 (viability/proliferation) |

| | |
|---|---|
| ceRNA | Competing endogenous RNA |
| CEu | Control eutopic endometrium |
| ChIP | Chromatin immunoprecipitation |
| circRNA | Circular RNA |
| Ctrl | Control |
| DE / DESeq2 | Differential expression / DESeq2 RNA-seq analysis package |
| DIA proteomics | Data-independent acquisition proteomics |
| DUSP6 | Dual-specificity phosphatase-6 |
| E-cad / N-cad | E-cadherin / N-cadherin |
| E2 | 17β-estradiol |
| EdU | 5-Ethynyl-2′-deoxyuridine (DNA synthesis assay) |
| EECs | Endometrial epithelial cells |
| EEsC / EESc | Ectopic endometrial stromal cells (patient-derived) |
| EMI | Endometrial–myometrial interface |
| EMT | Epithelial–mesenchymal transition |
| ER / ERα | Estrogen receptor / Estrogen receptor alpha |
| ERK1/2 | Extracellular signal-regulated kinases 1/2 |
| ESCs / hESCs | (Human) endometrial stromal cells |
| EV(s) | Extracellular vesicle(s) |
| FISH / IF | Fluorescence in situ hybridization / Immunofluorescence |
| fx/fx | Floxed allele (conditional) |
| GEO | Gene Expression Omnibus |
| GO / KEGG / Reactome | Gene Ontology / Kyoto Encyclopedia of Genes and Genomes / Reactome pathway databases |
| H&E / Masson | Hematoxylin–eosin / Masson's trichrome staining |
| H3K27me3 | Histone H3 lysine-27 trimethylation |
| HIF-1α | Hypoxia-inducible factor-1 alpha |
| ICAM1 / ITGAL | Intercellular adhesion molecule-1 / Integrin alpha-L |

| | |
|---|---|
| IHC | Immunohistochemistry |
| IPA | Ingenuity Pathway Analysis |
| ISH | In situ hybridization |
| JAK2/STAT3 | Janus kinase-2 / Signal transducer and activator of transcription-3 |
| JZ | Junctional zone |
| JZ-SMCs | Junctional-zone smooth muscle cells |
| KD / OE / sh / si | Knockdown / Overexpression / short hairpin RNA / small interfering RNA |
| KLF5 / KLF12 | Krüppel-like factor-5 / -12 |
| KO | Knockout |
| LASP1 | LIM and SH3 domain protein-1 |
| LH+2 / LH+7 | Days after LH surge (cycle phase stamps) |
| LIN28B | RNA-binding protein LIN28B |
| lncRNA | Long non-coding RNA |
| MAPK1 (ERK2) | Mitogen-activated protein kinase-1 |
| MGISEQ-2000 | High-throughput sequencing platform |
| miR / miRNA | MicroRNA |
| MMP-2 / MMP-9 | Matrix metalloproteinase-2 / -9 |
| MSP | Methylation-specific PCR |
| MTT | Tetrazolium viability assay |
| N-eMSCs | Normal endometrial mesenchymal stem cells |
| NF-κB | Nuclear factor kappa-B |
| NRP1 / NRP2 | Neuropilin-1 / -2 |
| NTA | Nanoparticle tracking analysis |
| p-PDK1 | Phosphorylated 3-phosphoinositide-dependent kinase-1 |
| P4 | Progesterone |
| PBAC | Pictorial blood loss assessment chart |
| PCA | Principal component analysis |
| PI3K–AKT–mTOR | Phosphoinositide-3 kinase–AKT–mammalian target of rapamycin pathway |

| | |
|---|---|
| PTEN | Phosphatase and tensin homolog |
| qPCR / RT-qPCR / ttRT-qPCR | (Reverse-transcription) quantitative PCR / two-tailed RT-qPCR |
| RIF | Recurrent implantation failure |
| RIP | RNA immunoprecipitation |
| RMA | Robust multi-array average (microarray normalization) |
| SLC15A4 | Solute carrier family 15 member A4 |
| STAT3 | Signal transducer and activator of transcription-3 |
| Talin1 (TLN1) | Cytoskeletal adaptor protein Talin-1 |
| TargetScan / miRDB / circBank / starBase / miRanda | ncRNA target/interaction prediction resources |
| TEM | Transmission electron microscopy |
| TGF-β1 | Transforming growth factor-β1 |
| ThESC | Telomerase-immortalized human endometrial stromal cell line |
| TLR4 | Toll-like receptor-4 |
| TSG101, CD9, CD63, CD81 | Canonical EV protein markers |
| UMIs | Unique molecular identifiers |
| VAS | Visual analog scale (symptom score) |
| VEGF | Vascular endothelial growth factor |
| Vim | Vimentin |
| WB | Western blot |
| Wnt/β-catenin (β-cat) | Wnt signaling / β-catenin |
| Wound-healing / Transwell | 2D migration assay / migration–invasion assay |
